# Short-term dynamics of input and output of CA1 network greatly differ between the dorsal and ventral rat hippocampus

**DOI:** 10.1186/s12868-019-0517-5

**Published:** 2019-07-22

**Authors:** Andriana Koutsoumpa, Costas Papatheodoropoulos

**Affiliations:** 1 Laboratory of Neurophysiology, Department of Medicine, University of Patras, 26504 Rion, Greece; 2 Present Address: Molecular Systems Biology, Groningen Biomolecular Sciences and Biotechnology Institute, University of Groningen, Groningen, The Netherlands

**Keywords:** Dorsoventral, Excitability, Hippocampus, Inhibition, In vitro, Mu opioid receptor, Rat, Septotemporal, Short-term plasticity, Short-term dynamics

## Abstract

**Background:**

The functional heterogeneity of the hippocampus along its longitudinal axis at the level of behavior is an established concept; however, the neurobiological mechanisms are still unknown. Diversifications in the functioning of intrinsic hippocampal circuitry including short-term dynamics of synaptic inputs and neuronal output, that are important determinants of information processing in the brain, may profoundly contribute to functional specializations along the hippocampus. The objectives of the present study were the examination of the role of the GABA_A_ receptor-mediated inhibition, the μ-opioid receptors and the effect of stimulation intensity on the dynamics of both synaptic input and neuronal output of CA1 region in the dorsal and ventral hippocampus. We used recordings of field potentials from adult rat hippocampal slices evoked by brief repetitive activation of Schaffer collaterals.

**Results:**

We find that the local CA1 circuit of the dorsal hippocampus presents a remarkably increased dynamic range of frequency-dependent short-term changes in both input and output, ranging from strong facilitation to intense depression at low and high stimulation frequencies respectively. Furthermore, the input–output relationship in the dorsal CA1 circuit is profoundly influenced by frequency and time of presynaptic activation. Strikingly, the ventral hippocampus responds mostly with depression, displaying a rather monotonous input–output relationship over frequency and time. Partial blockade of GABA_A_ receptor-mediated transmission (by 5 μM picrotoxin) profoundly influences input and output dynamics in the dorsal hippocampus but affected only the neuronal output in the ventral hippocampus. M-opioid receptors control short-term dynamics of input and output in the dorsal hippocampus but they play no role in the ventral hippocampus.

**Conclusion:**

The results demonstrate that information processing by CA1 local network is highly diversified between the dorsal and ventral hippocampus. Transient detection of incoming patterns of activity and frequency-dependent sustained signaling of amplified neuronal information may be assigned to the ventral and dorsal hippocampal circuitry respectively. This disparity should have profound implications for the functional roles ascribed to distinct segments along the long axis of the hippocampus.

**Electronic supplementary material:**

The online version of this article (10.1186/s12868-019-0517-5) contains supplementary material, which is available to authorized users.

## Background

Brain neuronal networks process information and perform a multitude of functions through highly dynamic activity states [1–3]. Rapid changes in neural activity can occur principally by dynamic modifications in the balance between excitation and inhibition [2, 4]. Synaptic plasticity, i.e. activity-dependent changes in synaptic effectiveness, can play a crucial role in modulation of excitation-inhibition relationship and neuronal input–output function thereby highly determining the state of activity in a neuronal network [5–8]. Importantly, dynamic changes in the activity of a network can endow the network with the ability of multiple modes of neural information processing which are assumed to be required in order to perform a variety of operations [1–3]. Very characteristically, the neural network of hippocampus displays a wide range of dynamic changes in its activity and performs a variety of functions by engaging different modes of activity [9, 10]. To some extent, this is expressed by the existence of different semi-independent nodes of neural information processing along a canonical excitatory trisynaptic circuit that defines the transverse “lamellar” organization of the hippocampus [11] and is composed by the dentate gyrus and the CA3 and CA1 fields that have their own properties and perform distinct operations [12, 13]. However, the many functions to which hippocampus plays important roles [14–17] may impose additional demands concerning the variety of neural computations of hippocampus needed to support these functions.

Indeed, the various hippocampal functions are not uniformly distributed along its longitudinal axis (see reviews [18–23]). This functional segregation is currently expressed most emphatically by the increased role of the dorsal hippocampus (DH) to encode spatial information and of the ventral hippocampus (VH) to process information related to emotionality [23, 24]. Though diversifications in external and internal hippocampal connectivity [25–28] can significantly support some aspects of behavioral diversification along the hippocampus, the fundamental operations performed by the hippocampal intrinsic circuitry [12, 16, 29] suggest that specializations of hippocampal network at the level of synaptic and cellular functioning along the longitudinal axis of the structure may also play crucial roles in supporting higher order functional segregation. During the last decades accumulating evidence shows that the functional organization of the hippocampal endogenous network present several-scale functional diversification along the long axis of the structure that may critically influence local information processing. For instance, differences have been found at the level of principal cell intrinsic excitability [30–34] and receptor function [35–39]; (see also recent reviews [21, 23, 40–43]).

Synaptic plasticity plays a major role in many hippocampal functions and the most extensively studied form is the phenomenon of long-term potentiation [44] mainly because of its robust conceptual link with long-term memory [45]. It is notable that the ability for induction of long-term potentiation and its neuromodulation greatly differs between the DH and the VH [37, 38, 42, 46–53] suggesting a distinct engagement of the two hippocampal segments to specific types of long-term memory.

Besides, hippocampus almost continuously receives highly integrated cortical information [29, 54–56], which must selectively and quickly be processed before some parts of it is long-term retained in the form of memory traces [57]. Virtually, the functions of fast processing (e.g. selection, comparison, integration etc.) could be accommodated by mechanisms of rapid network operations. Short-term forms of synaptic plasticity appear to play important roles in rapid neural information processing as recent experimental and modeling evidence suggest. Thus, short-term synaptic plasticity may serve the functions of temporal filtering, activity pattern detection, optimization of information transfer, dynamic gain control, stabilization of network activity and synaptic input diversification [58–65]. Therefore, the properties of short-term synaptic plasticity can effectively satisfy the requirements for rapidity and selectivity in information processing performed by hippocampal neuronal circuit. In addition, phenomena of short-term synaptic plasticity may be crucial in determining whether or not they will be followed by induction of long-term plastic changes [47, 66–69]. Furthermore, recent data point to an important role that short-term plasticity at hippocampal synapses can play to route activity propagation to extrahippocampal regions [70].

It has been recently shown that short-term plasticity of CA3 to CA1 synaptic connections displays a frequency-dependent gradually diversified pattern of responses along the longitudinal axis of the hippocampus [71]. Accordingly, the striking differences in short-term synaptic plasticity that occur along the hippocampus may drastically influence the output of CA1 neural circuitry since an important consequence of synaptic plasticity is the modulation of input–output relationship in a network [65]. However, how the spiking activity of CA1 neurons is influenced by frequency-depended short-term synaptic plasticity over the dorsoventral hippocampal axis is unknown. In addition to synaptic plasticity, other mechanisms also contribute to determining input–output relationship. These mechanisms may importantly include synaptic inhibition [5, 72]. However, whether and how synaptic inhibition is involved in shaping dorsal–ventral differences in short-term dynamics in the hippocampus is not known.

The objectives of the present study were to systematically assess (a) the dynamic changes produced by short-lasting repetitive presynaptic activation of varying frequency and intensity in the synaptic transmission and neuronal excitation in the DH and VH CA1 hippocampal circuitry, (b) the actions of GABA_A_ receptor- and μ opioid receptor-mediated transmission in short-term dynamics of the two hippocampal segments. In order to achieve these objectives we have used recordings of evoked field potentials from transverse hippocampal slices obtained from adult male rats. The transverse slice obtained from the rodent hippocampus is the most widespread preparation used for the study of synaptic plasticity [73, 74]. Furthermore, a growing body of experimental data is being accumulated on physiological specializations along the longitudinal axis of rat hippocampus, thereby facilitating interpretations of the present data.

We find that short-term dynamics of both synaptic input and neuronal spiking activity, which depend on the strength of presynaptic activation, greatly differ between the DH and VH. Remarkably, GABAergic inhibition profoundly controls short-term synaptic plasticity in the DH only, though it controls spiking activity in both segments, and μ-ORs modulate short-term dynamics in the DH but not the VH.

## Methods

### Animals and hippocampal slice preparation

Thirty one male Wistar rats (RRID:RGD_10028) 3 to 4 months old are used in this study. Rats were maintained under stable conditions of light–dark cycle (12/12 h), temperature (20–22 °C) and they had free access to food and water, at the SPF Laboratory of Experimental Animals of the Department of Medicine, University of Patras (licence No: EL-13-BIOexp-04). Two adult rats were kept in each cage, according to the instructions provided. Experiments were conducted in accordance with the European Communities Council Directive Guidelines for the care and use of Laboratory animals (2010/63/EU-European Commission) and they have been approved by the “Protocol Evaluation Committee” of the Department of Medicine of the University of Patras and the Directorate of Veterinary Services of the Achaia Prefecture of Western Greece Region (reg. number: 203173/1049, 22/08/2014). Furthermore, this manuscript reporting adheres to the ARRIVE guidelines for the reporting of animal experiments. In addition, we have made every effort to minimize the number of animals used. In particular, we have used the statistical power analysis program G*power 3.1.9.2 to a priori compute the number of rats required in this study. Each day, a rat was randomly selected from the colony according to a simple randomization method [75]. Each rat corresponded to an electrophysiology experiment which started at 9:00–10:00 a.m. Each day, a rat was transferred from the Laboratory of Experimental Animals to the laboratory of Neurophysiology for scarification. The animal was positioned inside a metallic case (~ 45 cm^2^ large) and deeply anaesthetized via the respiratory route by exposure to ~ 15 ml diethyl-ether for approximately 2 min with continuous monitoring of heart rate. Rat was decapitated when heart rate was reduced to approximately one beat per second. Following decapitation, the cranium was opened by three incisions and the brain was removed and placed in ice-cold (2–4 °C) standard artificial cerebrospinal fluid (ACSF) containing, in mM: 124 NaCl, 4 KCl, 2 CaCl_2_, 2 MgSO_4_, 26 NaHCO_3_, 1.25 NaH_2_PO_4_ and 10 glucose. ACSF was equilibrated with 95% O_2_ and 5% CO_2_ gas mixture at a pH = 7.4. Under these conditions each hippocampus was excised free from the brain and placed on the disc of a McIlwain tissue chopper. Transverse 500–550 µm-thick slices were prepared from the dorsal (septal) and the ventral (temporal) segment of the hippocampus extending between 1.0 mm and 3.0 mm from the DH and the VH end, as previously described [52, 76]. Immediately after sectioning, slices were transferred to an interface type recording chamber where they were maintained continuously perfused with fresh ACSF of the same composition as above described at a rate of ~ 1.5 ml/min. Slices were continuously humidified with a mixed gas consisting of 95% O_2_ and 5% CO_2_ at a constant temperature of 30 ± 0.5 °C. Tissue stimulation and recording started at least one and a half hours after their placement in the chamber. The blocker of GABA_A_ receptor-associated channel picrotoxin (PTX, 5 μM) and the specific agonist of μ opioid receptors *N*-phenyl-*N*-[1-(2-phenylethyl)-4-piperidinyl]propanamide citrate salt (fentanyl, 10 μΜ) were used; both substances were purchased from Sigma-Aldrich, Germany.

### Electrophysiological recordings, processing and data analysis

Population potentials were evoked by electrical stimulation of the Schaffer collaterals using a wire-made bipolar platinum/iridium electrode with a wire diameter of 25 μm and an inter-wire distance of 100 μm (World Precision Instruments, USA). Field excitatory postsynaptic potentials (fEPSPs) and population spikes (PS) were recorded from the middle stratum radiatum and stratum pyramidale of the CA1 field respectively using a 7 μm-thick carbon fiber (Kation Scientific, Minneapolis, USA) positioned 350 μm from the stimulation electrode. When both fEPSP and PS were recorded from an individual slice, they were recorded simultaneously. Electrical stimulation consisted of current pulses of a fixed duration (100 μs) and variable amplitude (20 to 260 μA). Baseline stimulation was delivered every 30 s using a current intensity that elicited a just-subthreshold fEPSP on the basis of input–output curves between stimulation intensity and evoked responses. Short-term changes in fEPSP and PS were studied using a frequency stimulation protocol that consisted of a sequence of ten consecutive pulses delivered at the following frequencies: 0.1, 1, 3, 5, 10, 20, 30, 40, 50, 75 and 100 Hz. The number of pulses falls into the range of naturally occurring spike trains in CA3 cells [77]. Consecutive trains of pulses, delivered during the application of frequency stimulation paradigm, were separated by a 2-min interval in order to allow synapses to return to baseline level. The frequency stimulation paradigm was applied at two or three intensities of stimulation current according to experimental requirements (see “Results”). A lapse of 5 min interspaced between consecutive experimental epochs of different stimulation current intensities. Signal was amplified 500 times and band-pass filtered at 0.5 Hz–2 kHz using Neurolog amplifiers (Digitimer Limited, UK), digitized at 10 kHz and stored on a computer disk for off-line analysis using the CED 1401-plus interface and the Signal6 software (Cambridge Electronic Design, Cambridge, UK). The fEPSP was quantified by the maximum slope of its initial rising phase; slope was measured in a time window of one millisecond immediately after the appearance of the presynaptic fiber volley. PS was quantified by its amplitude measured by the length of the projection of the minimum peak on the line connecting the two maxima peaks of the PS waveform. The effects of frequency stimulation were quantified as the percent change of each of the nine consecutive responses evoked by the consecutive pulses with respect to the first response in the train. Steady-state response was estimated by averaging the last three responses (i.e. 8th–10th).

The following tests were used for statistical comparisons: paired and independent t-tests, multivariate general linear model (MANOVA), one-way analysis of variance (ANOVA) and bivariate correlation analysis. The IBM SPSS software package was used for all statistical analyses, including the methods used to assess whether the data met the assumptions of particular statistical approach performed during the study. The values in the text and figures express mean ± SEM. The number of slices and animals used in the analysis (slices/animals) is given throughout the text. The statistics were performed using the number of slices. On average, two slices from individual rats were used to prepare statistics.

## Results

### Stimulation strength influences short-term synaptic plasticity more in DH than in VH

**Fig. 1:**
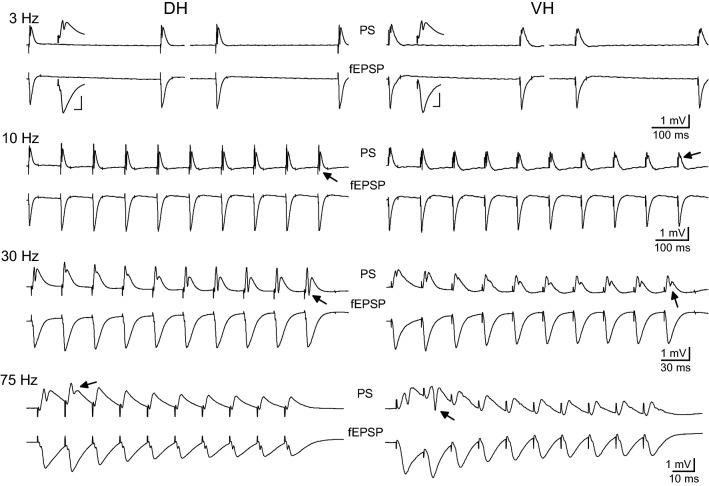
Examples of evoked responses from the CA1 stratum radiatum (fEPSPs) and stratum pyramidale (PS) recorded following the delivery of a ten-pulse suprathreshold stimulation train at Schaffer collaterals in dorsal and ventral hippocampal slices. Traces obtained at four representative stimulation frequencies are shown. Only the two first and the two last responses evoked by 3 Hz stimulation are shown for clarity reasons. Stimulation intensity was adjusted to produce a suprathreshold fEPSP that induced a PS of 0.5–1 mV amplitude. Single traces shown on the top and bottom of PS and fEPSP traces of 3 Hz respectively, in each panel, represent the first response of the 0.1 Hz train with which each frequency stimulation experimental procedure started; calibration bars: 1 mV, 5 ms. Stimulation artifacts are truncated for clarity. Arrows indicate the different affects of frequency stimulation on PS between DH and VH

**Fig. 2:**
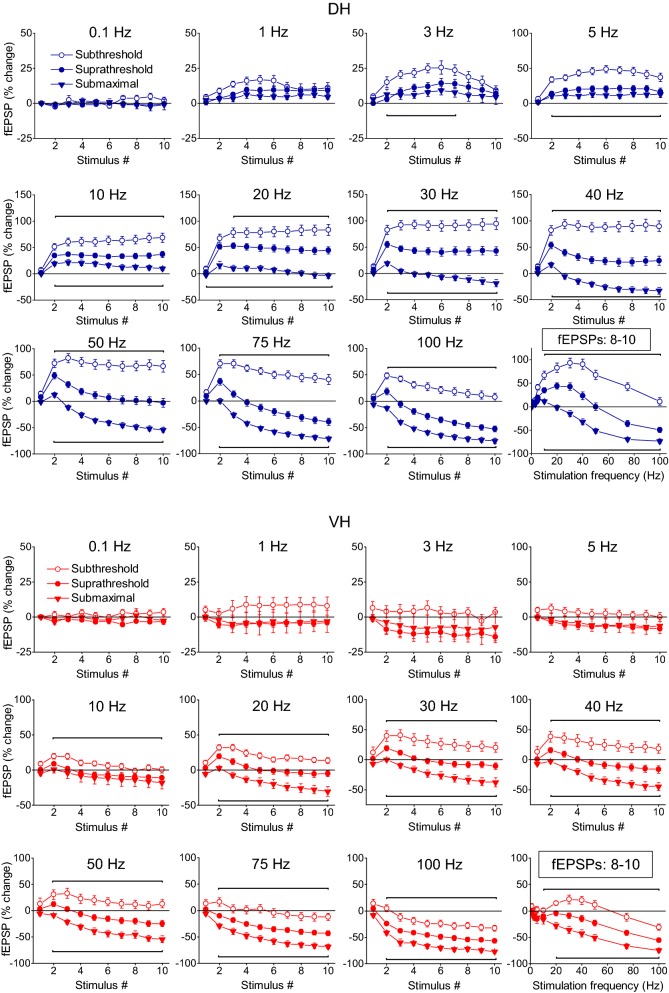
Short-term synaptic plasticity (fEPSP) depends on stimulation intensity and highly differs between the DH and the VH. Short-term changes in fEPSP induced by a ten-pulse train delivered at three stimulation intensities (subthreshold, suprathreshold and submaximal synaptic responses indicated by open circles, filled circles and diamonds respectively) applied at stimulation frequencies from 0.1 to 100 Hz are shown for the DH and the VH (upper and lower panel respectively). In all diagrams but the two bottom right graphs, fEPSP changes are plotted as a function of stimulus number. The bottom right diagrams in the two panels show the average value of fEPSP changes produced by the 8th–10th stimuli (steady state response) plotted as a function of stimulation frequency. The intensity of stimulation current significantly affected steady-state changes of fEPSP at 5–100 Hz in DH (one-way ANOVA across stimulation intensity, from a minimum F = 8.76, *p *< 0.005 at 5 Hz to a maximum F = 69.9, *p *< 0.001 at 50 and 75 Hz). Similarly, stimulation current intensity significantly affected steady-state changes of fEPSP at 10-100 Hz in VH (one-way ANOVA across stimulation intensity, from a minimum F = 4.1, *p *< 0.05 at 10 Hz to a maximum F = 22.25 at 20 Hz; *p *< 0.001). Also, the results of statistical comparison of all responses evoked along the stimulation train by the three different stimulation current intensities are shown by horizontal bars (MANOVA along the entire train of responses and independent t-test of individual responses along the train, *p *< 0.05). Specifically, bars above and below the data curves show the stimulation epoch in which significantly different responses were observed between subthreshold and suprathreshold stimulation (bars above the curves) and suprathreshold and submaximal stimulation (bars below the curves). Note that steady-states of fEPSP changes (i.e. averages of 8–10th responses) shown in bottom-right graphs in the two panels, greatly differ between DH and VH especially when evoked by subthreshold and suprathreshold stimulation intensities (see also Fig. 3). Data presented in the three stimulation intensities (subthreshold, suprathreshold and submaximal) were obtained from (slices/rats): 10/5, 11/6 and 9/4 in DH and 11/6, 11/6 and 8/5 in VH

We first examined the effects of the intensity of presynaptic stimulation on short-term dynamics of both input (i.e. fEPSP) and output (i.e. PS) of CA1 hippocampal microcircuit. It has been recently reported that the short-term synaptic plasticity in CA1 greatly differs along the dorsoventral hippocampal axis when examined at a stimulation intensity producing a just-subthreshold fEPSP [71]. However, it is known that the short-term synaptic plasticity is inversely related to the intensity of presynaptic activation [78, 79]. Thus, we wondered whether the large dorsoventral differences in short-term synaptic plasticity observed at moderate stimulation intensity also exist at lower and higher levels of presynaptic activation. Furthermore, because fluctuations in the strength of synaptic input are expected to drastically influence neuronal firing [80] we also aimed to study whether and how the neuronal excitation is related to short-term synaptic plasticity at different activation levels. For this purpose we chose to apply frequency stimulation (consisted of a ten-pulse train) at three different stimulation intensities that produced either subthreshold responses or postsynaptic potentials that triggered action potentials. Specifically, we adjusted the stimulation current intensity to alternatively produce: (a) a subthreshold fEPSP (0.34 ± 0.02 mV/ms); (b) a suprathreshold fEPSP (1.27 ± 0.08 mV/ms) that evoked a PS of 0.5–1.0 mV (0.71 ± 0.05 mV); (c) a PS of 75% of its maximum amplitude (4.05 ± 0.3 mV, and a corresponding fEPSP of 2.55 ± 0.25 mV/ms). The three stimulation intensities that produced three different levels of local network activation will be thereafter called subthreshold, suprathreshold and submaximal, respectively. We applied this experimental protocol in DH and VH slices obtained from six rats. Examples of responses evoked by suprathreshold stimulation are shown in Fig. 1. The numbers of slices/animals used in this experimental protocol are given in the legend of Fig. 2.

We observed stimulation-dependent and frequency-dependent changes in fEPSP recorded in either hippocampal segments; however, these changes were much more robust in DH compared with VH slices. In particular, we find that the intensity of presynaptic stimulation significantly influences fEPSPs elicited along the ten-pulse train at stimulation frequencies between 5 and 100 Hz in the DH and at stimulation frequencies between 10 and 100 Hz in the VH (MANOVA inside each stimulation frequency and along the stimulation train, in DH and VH). More details of statistical analysis are given in the legends of Fig. 2).

**Fig. 3:**
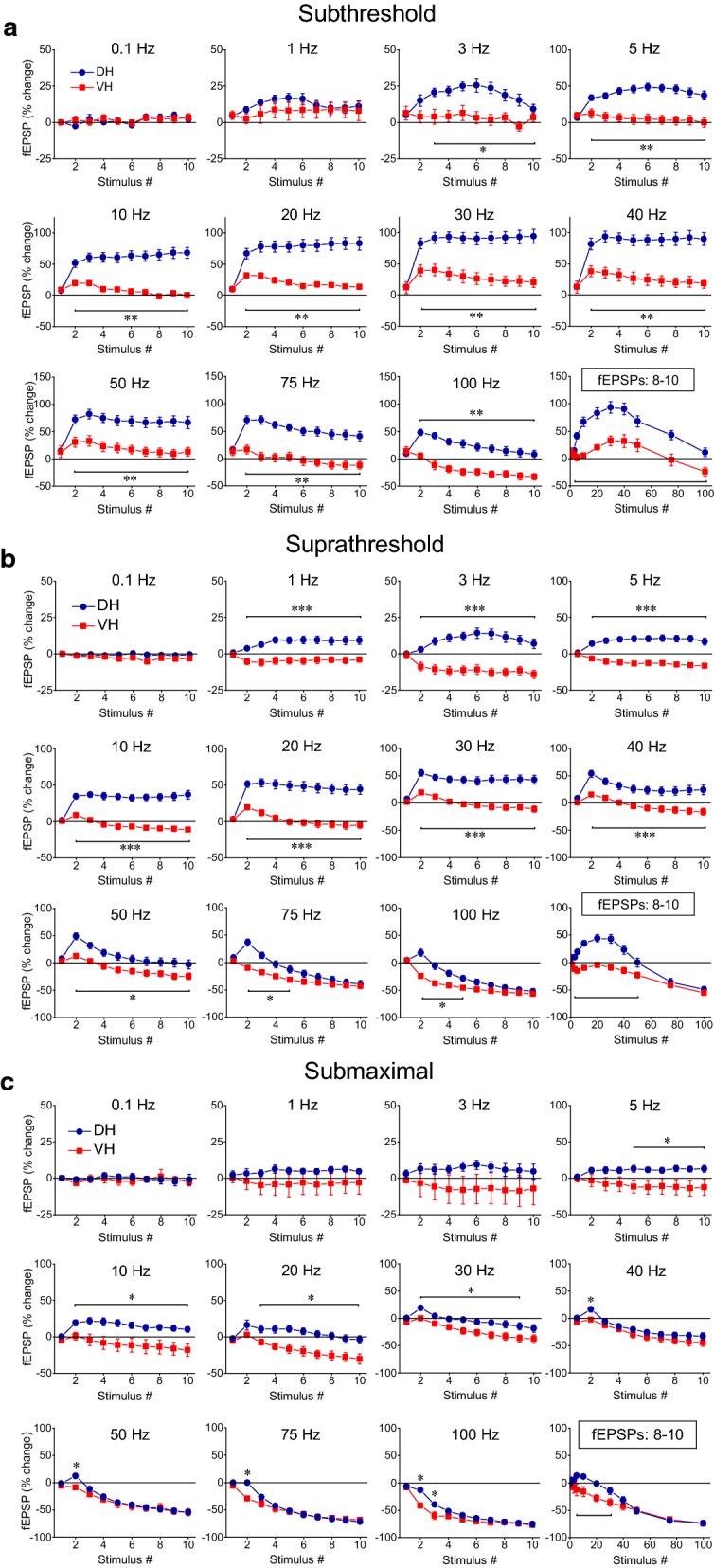
Short-term dynamics of fEPSPs in DH and VH. Data presented in this figure are the same as the data presented in Fig. 2, but here values obtained from DH (circles) and VH (squares) are plotted on the same graphs for facilitating comparisons between the two hippocampal segments. Data obtained with the three stimulation current intensities, i.e. subthreshold, suprathreshold and submaximal are shown in **a**–**c** respectively. Statistically significant differences of responses evoked along the entire stimulation train, between the two hippocampal segments are shown by horizontal bars (MANOVA along entire train of responses and independent t-test of individual responses along the train, *p < 0.05, **p < 0.01, ***p < 0.005). Note that changes of fEPSPs induced by the stimulation train, including steady-states of fEPSP changes (i.e. averages of 8–10th responses, bottom-right graphs in each panel) significantly differ between DH and VH at 3–100 Hz with subthreshold stimulation, 1–50 Hz at suprathreshold stimulation and 5–30 Hz with submaximal stimulation. The frequency range of statistical significance of dorsoventral differences in the three graphs of steady-state responses is marked by a horizontal bar without asterisk. Note that steady-state depression induced by high-frequency suprathreshold and submaximal stimulation (at 75–100 Hz and 40–100 Hz respectively) did not significantly differ between the two hippocampal segments (independent t-test, *p *> 0.05)

The DH showed a robust steady-state facilitation of fEPSP with subthreshold stimulation (90–95% at 30–40 Hz) and suprathreshold stimulation (40–45% at 20–30 Hz), while submaximal stimulation produced a moderate steady-state facilitation at low frequencies (1–10 Hz) and a robust steady-state depression (70–75%) at high frequencies (75–100 Hz), (Fig. 2, DH). More specifically, subthreshold stimulation in the DH produced a significant steady-state facilitation of fEPSP at stimulation frequencies 1–75 Hz (paired *t* test between the baseline fEPSP and the average of 8–10th fEPSPs, *p *< 0.05). Suprathreshold stimulation in the DH produced significant steady-state facilitation at 1–40 Hz and significant depression at 75–100 Hz while submaximal stimulation produced significant steady-state facilitation at 5–10 Hz and depression at 30–100 Hz (paired t-test between the first fEPSP and the average of 8–10th fEPSPs, *p *< 0.05), (Fig. 2, DH). On the contrary, the VH presented a very different pattern of short-term changes in fEPSP (Figs. 1, 2, VH). Steady-state facilitation of fEPSP in the VH occurred only with subthreshold stimulation at a narrow range of stimulation frequencies (20-40 Hz) and maximum values amounted only 15–20% (paired t-test between the baseline fEPSP and the average of 8–10th fEPSPs, *p *< 0.05). Suprathreshold and submaximal stimulation of VH produced only a steady-state depression of fEPSP at a wide range of stimulation frequencies (5–100 Hz) with maximum values obtained at high stimulation frequencies (75–100 Hz, 40–75%; paired t-test for each stimulation frequency, *p *< 0.05). Therefore, the synapses of the DH display an enhanced range of short-term changes of fEPSP, from robust steady-state facilitation induced by subthreshold stimulation of moderate frequency (20–40 Hz) to strong steady-state depression produced by high-frequency (50–100 Hz) submaximal stimulation (Figs. 2, 3). On the contrary, VH synapses display a moderate facilitation of fEPSP with subthreshold stimulation and a steady-state depression with stronger stimulation intensities (Figs. 2, 3). Accordingly, robust dorsoventral differences in short-term synaptic plasticity were found in the present study especially when subthreshold and suprathreshold stimulation intensities were used (Fig. 3a, b). Specifically, we found significant dorsoventral differences in steady-state fEPSP changes across a wide range of frequencies of subthreshold and suprathreshold stimulation (at 3–100 Hz and 1–50 Hz respectively, MANOVA, *p *< 0.01; for more details see the legend of Fig. 3). Different steady-state changes of fEPSP in DH and VH were also observed with submaximal stimulation at frequencies 5–30 Hz (Fig. 3c). However, similar amounts of steady-state depression elicited in the two hippocampal segments by high-frequency suprathreshold stimulation (75–100 Hz) and submaximal stimulation (at 0.1–3 Hz and 40–100 Hz). For additional statistics and the number of slices/rats used see Figs. 2 and 3. Also, individual data points for steady-state changes in fEPSP are presented in scatter plots of panel a in Additional file 1: Figure S1.

### Neuronal output can be highly amplified in the DH but mostly depressed in the VH

**Fig. 4:**
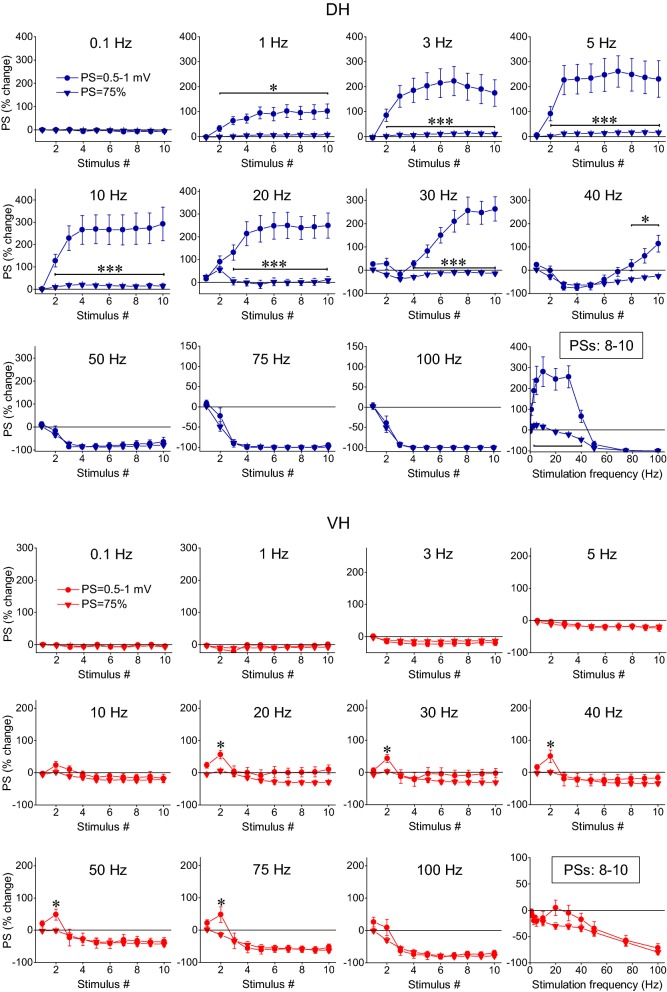
Short-term dynamics of neuronal output (PS) depends on stimulation frequency and intensity and highly differ between DH and VH. Diagrams in the two panels (upper, DH and lower, VH) show frequency-dependent short-term changes in PS induced by a ten-pulse train delivered at two stimulation intensities producing a PS of 0.5–1 mV (suprathreshold response; circles) and a PS 75% of its maximal value (submaximal response; diamonds) respectively. The bottom right diagrams in the two panels show the average value of PS changes produced by the 8th–10th stimuli (steady state response) plotted as a function of stimulation frequency. Horizontal bars mark the stimulation epoch in which significantly different responses were observed between suprathreshold and submaximal stimulation (MANOVA among all responses in a train and independent t-test of individual responses). Asterisks denote the level of significance: *p < 0.05, ***p < 0.005. Data presented in the two stimulation intensities (suprathreshold and submaximal) were obtained from (slices/rats): 11/6 and 9/4 in DH and 11/6, and 10/5 in VH. In the DH the intensity of 1–40 Hz stimulation strongly influenced steady-state changes of PS (independent t-test, bottom-right graph). On the contrary, only the second response in a train was significantly facilitated by 20–75 Hz suprathreshold stimulation in the VH (MANOVA and independent t-test). Stimulation intensity did not significantly influence steady-states of PS in the VH at any stimulation frequency (independent t-test, *p *> 0.05). Note that steady-states of PS changes greatly differ between DH and VH (see also Fig. 5)

For the study of the short-term dynamics of neuronal output, measured by PS, we used two stimulation current intensities, suprathreshold and submaximal, as preliminary experiments showed that a (initially) subthreshold stimulation does not trigger a PS in any stage or frequency of stimulation. This experimental protocol was applied in DH and VH slices obtained from six rats. More details on the number of slices/animals are given in the legend of Fig. 4. We find that the stimulation train caused strikingly different patterns of PS changes between DH and VH and these changes strongly depended on the stimulation intensity in the DH but not in the VH (Figs. 1, 4). In general, the changes in PS recorded from the DH ranged from strong steady-state facilitation (240–300% caused by 5–30 Hz of suprathreshold stimulation) to steady-state complete depression (100%, with 75–100 Hz stimulation of either intensity). In sharp contrast, the steady-state output response of VH was generally a non complete depression, which amounted to a maximum of 80% with submaximal stimulation. Yet, neuronal output in VH transiently facilitated at the beginning of suprathreshold stimulation at stimulation frequencies of 30–100 Hz (Fig. 4, VH).

In particular, 1–40 Hz suprathreshold and submaximal stimulation in the DH produced very different steady-state changes of PS (MANOVA across the stimulation train and independent t-test of steady-state responses between the two stimulation intensities; detailed statistics are given in Fig. 4). More specifically, subthreshold stimulation delivered at 1–30 Hz caused a strong steady-state facilitation of PS (paired t-test between the baseline PS and the average of 8–10th PSs, *p *< 0.005). Interestingly, at 30 Hz there is an initial transient and moderate reduction in PS (20%), which, however, transformed into strong steady-state facilitation (255%). At 40 Hz the initial depression of PS was robust (with a maximum of 75%), lasted longer (3–7th responses in the train), and was followed by significant facilitation (that amounted 20–117% during the 8–10th responses). Stimulation at 50 Hz caused robust steady-state depression of PS (by 80%) and a complete suppression of PS produced by stimulation at 75–100 Hz virtually blocked the output of the network (paired t-test at stimulation frequencies 50–100 Hz, *p *< 0.001) (Fig. 4, DH). Accordingly, the frequency range of 30–50 Hz marks an abrupt transition phase in the behavior of the DH network acting as a converter between facilitation and depression of neuronal spiking activity. Submaximal stimulation produced only a small yet significant steady-state facilitation of PS at stimulation frequencies of 1–5 Hz (7–17%, paired t-test, *p *< 0.01), no significant change at 10–30 Hz (paired t-test, *p *> 0.05) and a significant steady-state depression of PS at stimulation frequencies between 40 and 100 Hz (40–100%, paired t-test between baseline PS and the average of 8–10th PSs, *p *< 0.001). Practically, the depression of PS produced at 50–100 Hz was similar in the two stimulation intensities (MANOVA and independent t-test between suprathreshold and submaximal stimulation, *p *> 0.05) (Fig. 4, DH). It is noted that in the DH the relationship between input and output is highly and dynamically changed along stimulation time and over the different stimulation frequencies (compare Fig. 2 with Fig. 4, DH).

**Fig. 5:**
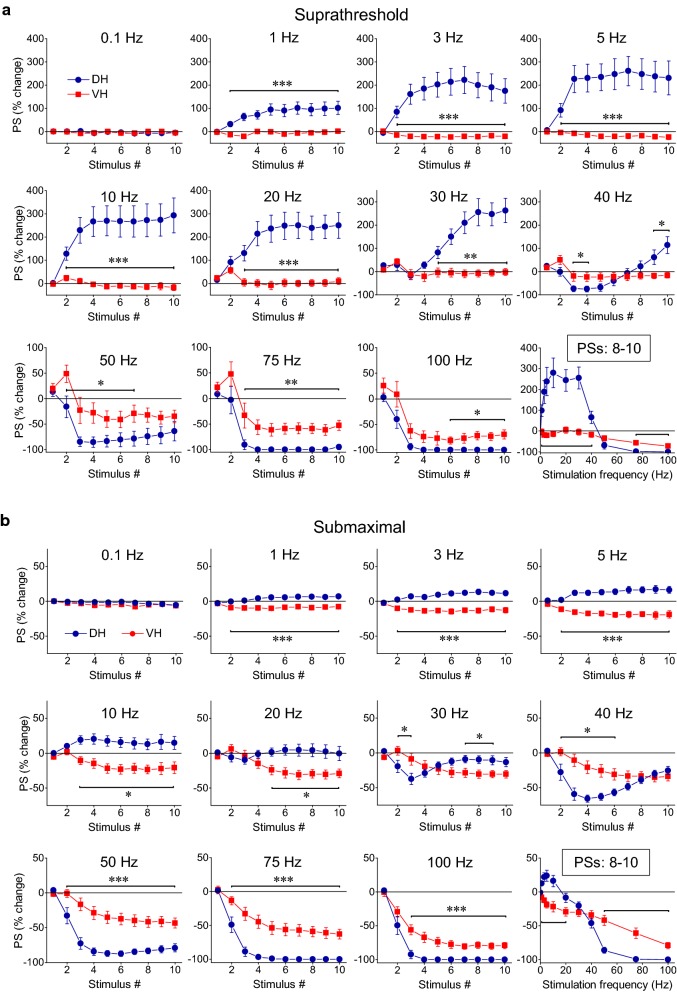
Short-term dynamics of PS in DH and VH. Data presented in this figure are the same as the data presented in Fig. 4, but here values obtained from DH (circles) and VH (squares) are plotted on the same graphs for facilitating comparisons between the two hippocampal segments. Data obtained with the two stimulation current intensities, i.e. suprathreshold and submaximal are shown in **a**, **b** respectively. The results of statistical comparison of all responses evoked along the entire stimulation train by the three different stimulation current intensities are shown by horizontal bars (MANOVA along the train of responses and independent t-test of individual responses along the train, *p < 0.05, **p < 0.01, ***p < 0.005). Note that most of the responses induced along the stimulation train significantly differ between the two hippocampal segments at almost all stimulation frequencies. Steady-state responses significantly differ between DH and VH at 1–40 Hz and 75–100 Hz with suprathreshold stimulation and at 1–20 Hz and 50–100 Hz with submaximal stimulation (horizontal bars in bottom right graphs)

**Fig. 6:**
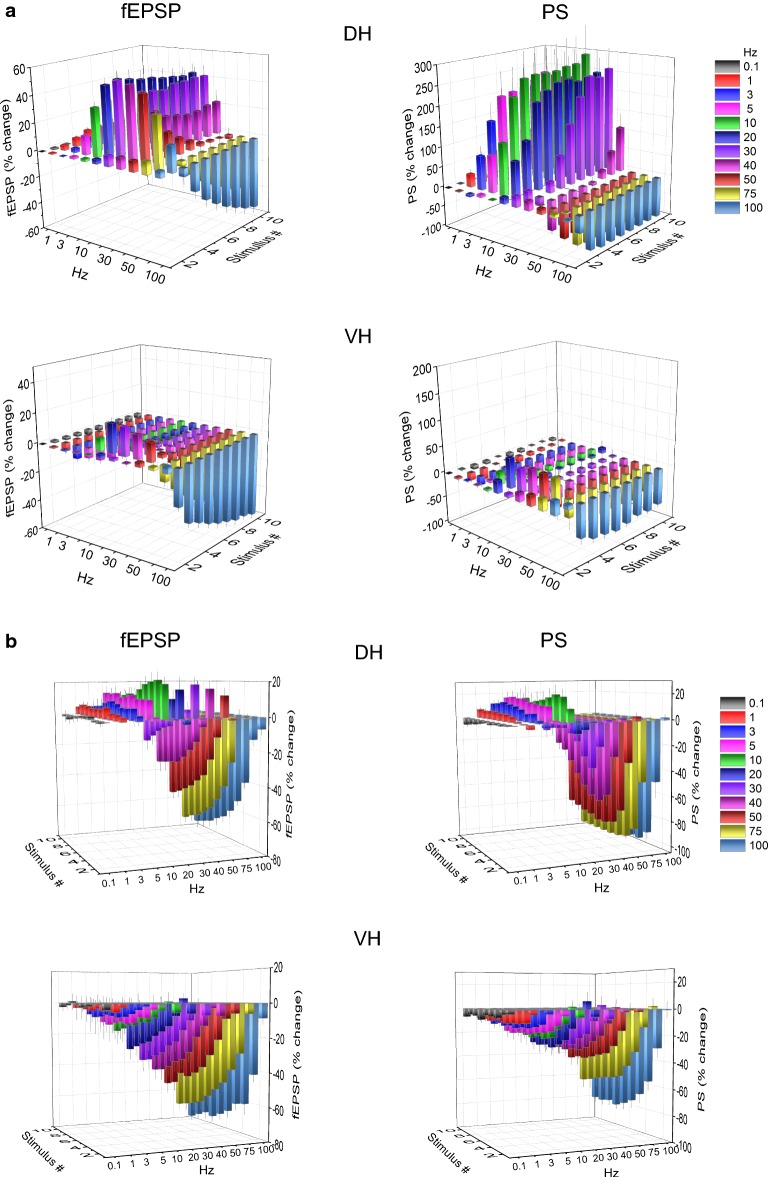
Summary figure describing the very different profiles of short-term dynamics for both synaptic input (fEPSP) and neuronal output (PS) between DH and VH. Collective 3D diagrams illustrating the short-term changes in synaptic transmission (fEPSP) and neuronal excitation (PS) induced by suprathreshold (i.e. PS = 0.5–1 mV) and submaximal stimulation (PS = 75% of maximal value) are shown in **a**, **b** respectively. Changes in fEPSPs and PSs are plotted as a function of stimulus frequency and the number of stimuli. Note that Y-axis scale is different in the graphs of fEPSP and PS in **a** for clarity reasons

**Fig. 7:**
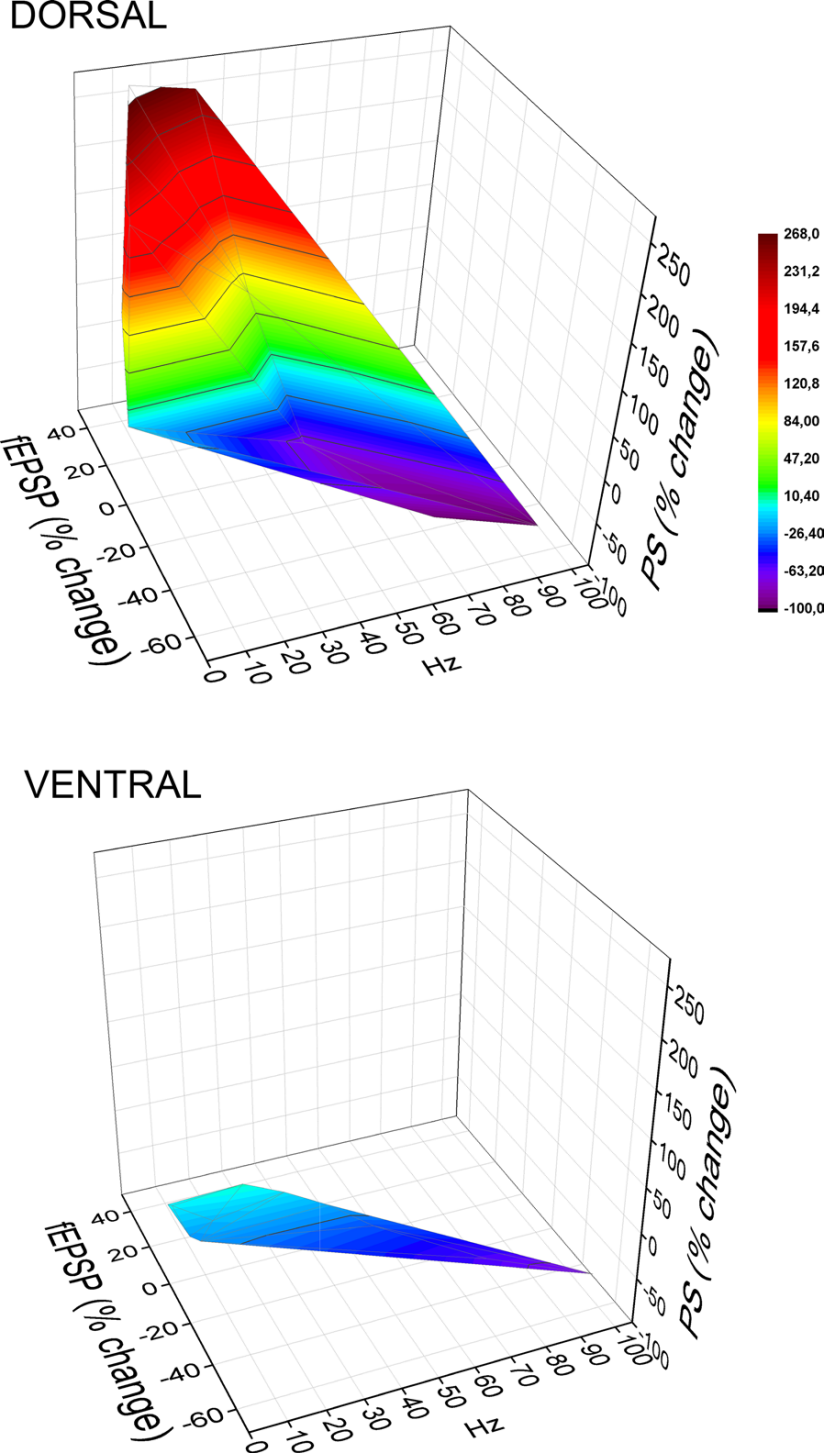
The dynamic range of frequency-dependent modulation of synaptic input (fEPSP) and neuronal output (PS) greatly differs between the dorsal and the ventral hippocampal circuitry. The two graphs show the combined steady-state changes in fEPSP and PS as a function of stimulus frequency. Data are obtained at suprathreshold stimulation intensity. The highest and the lowest value of each 3D plot (corresponding to intense facilitation and depression respectively) are marked by red and blue color respectively, as indicated in the color scale bar. In the DH the changes of both fEPSP and PS range from robust facilitation to almost complete depression. On the contrary, the steady-state responses in the VH (both fEPSP and PS) are mostly depressed, showing only a minor facilitation at low stimulus frequency

In the VH, frequency stimulation caused a steady-state depression of PS which was linearly correlated with increasing stimulation frequency (bivariate correlation, r = − 0.493, *p *< 0.001 and r = − 0.778, *p *< 0.001 for suprathreshold and submaximal stimulation respectively) (Fig. 4, VH). Suprathreshold stimulation consistently produced a significant steady-state depression of PS at high stimulation frequencies (50–100 Hz) as well as at low frequencies (3–5 Hz) (paired t-test between the baseline and steady-state response at each stimulation frequency, *p *< 0.05). Moreover, submaximal stimulation consistently caused a steady-state depression of PS at all stimulation frequencies from 0.1 to 100 Hz (paired t-test, *p *< 0.05). It is noted that the neuronal output in the VH was not completely blocked at high-frequency stimulation (75–100 Hz) as occurred in DH. Also, PS was very transiently and significantly facilitated at the beginning of suprathreshold stimulation applied at frequencies 20–75 Hz (paired t-test, *p *< 0.05) (Fig. 4, VH). Comparison of neuronal output dynamics between DH and VH demonstrated that they are very different between the two hippocampal segments and in relation of stimulation intensity and stimulation frequency (Fig. 5). Thus, as shown in Fig. 5a, suprathreshold stimulation applied at 1–100 Hz produced significantly different steady-state changes of PS between the DH and the VH (independent t-test, *p *< 0.005). In particular, at stimulation frequencies 1–40 Hz the DH displayed strong steady-state facilitation while the VH displayed steady-state depression. Moreover, the depression of neuronal output observed at 50-100 Hz was significantly greater in DH than in VH (MANOVA and independent t-test; details of statistical comparison are given in Fig. 5). Furthermore, with submaximal stimulation (Fig. 5b) the steady-state changes of PS differed between the two hippocampal segments for stimulation frequencies 1–20 Hz (facilitation or not change in DH and depression in VH) and 50–100 Hz (greater depression in DH than in VH) (MANOVA and independent t-test, for detailed statistical results see Fig. 5). Individual data points for steady-state changes in PS are presented in scatter plots of panel b in Additional file 1: Figure S1. Given that the PS response was strongly facilitated only in the DH and that was completely depressed in the DH but not the VH, it is concluded that the dynamic range of frequency-dependent modulation of neuronal output is wider in the DH than in the VH (Figs. 6, 7). Furthermore, comparisons of short-term dynamics between input and output showed striking differences between DH and VH. Thus, the short-term dynamics of PS observed with suprathreshold stimulation differ from those of fEPSP in the DH (compare Fig. 2 with Fig. 4, DH) whereas the pattern of changes of PS and fEPSP induced by either stimulation intensity (i.e. suprathreshold or submaximal) are quite similar in VH (compare Fig. 2 with Fig. 4, VH). For instance, with 30 Hz of suprathreshold stimulation the modulation of output in the DH is strongly biphasic (i.e. initial depression is followed by intense facilitation during stimulation) despite a stable level of moderate facilitation of synaptic input, while both synaptic input and neuronal output show a similar pattern in VH. All synaptic and neuronal responses (fEPSP and PS respectively) induced by suprathreshold and submaximal ten-pulse stimulation and at 0.1–100 Hz are presented in a summary figure (Fig. 6) (in summary Fig. 6). Furthermore, the wider dynamic range of frequency-dependent changes in fEPSP and PS induced by suprathreshold stimulation observed in the DH compared with the VH is illustrated in Fig. 7.

### Inhibition differently controls short-term-dynamics in the DH and the VH

The above described results suggested that the mechanisms of short-term facilitation of input and output are especially effective and powerfully frequency-dependent in the DH but they are moderate and depend much less on the stimulation frequency in the VH. In addition, some data suggests that input and output are modulated by partly different mechanisms. For instance, especially suggestive of mechanistic distinction between input and output modulation is the conspicuously different pattern of fEPSP and PS changes induced by suprathreshold 30 Hz stimulation in DH.

There are several proposed mechanisms that may underlie short-term synaptic plasticity, many of them being presynaptic in nature [81, 82]. In addition, postsynaptic inhibition may play a significant role as recent experimental results had shown [83, 84]. Thus, besides the fact that short-term dynamics of neuronal excitation may significantly rely on short-term synaptic plasticity, inhibition may also play a particularly important role on determining neuronal output dynamics [85, 86]. In addition, the intensity of presynaptic stimulation has a profound effect on neuronal excitation by affecting, for instance, the degree of activation of local networks of inhibitory neurons [87]. Therefore, we asked whether and how synaptic inhibition is involved in modulating short-term dynamics of input and output of CA1 microcircuit in the DH and the VH.

**Fig. 8:**
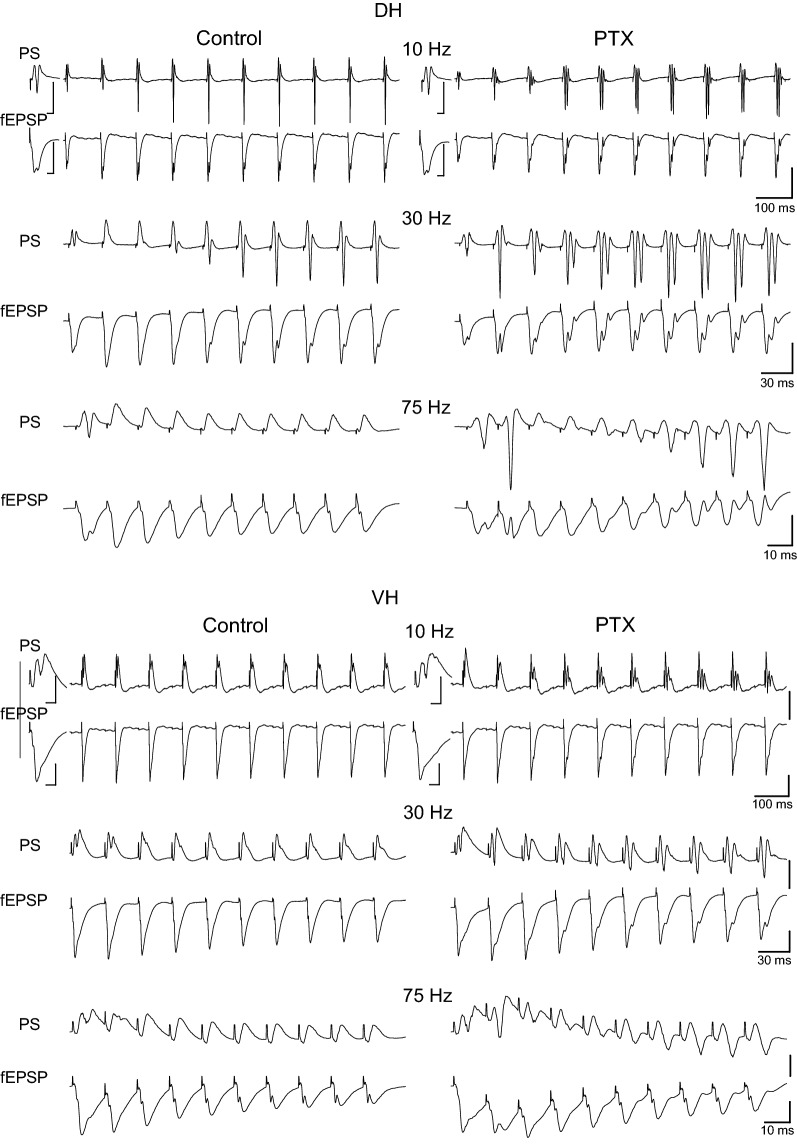
Examples of recordings of fEPSPs and PS evoked by three representative frequencies of stimulation in a DH and a VH slice. Traces were obtained before and during application of the blocker of GABA_A_ receptor channel picrotoxin (open and filled symbols respectively). Stimulation intensity was adjusted to produce a suprathreshold fEPSP that induced a PS of 0.5–1 mV amplitude. Single traces on the left of first PS and fEPSP traces (i.e. in the 10 Hz example) in each panel represent the first response of the 0.1 Hz train with which each frequency stimulation experimental procedure started; time calibration bars, 5 ms. Amplitude calibration bar for all records, 1 mV. Stimulation artifacts are truncated for clarity

**Fig. 9:**
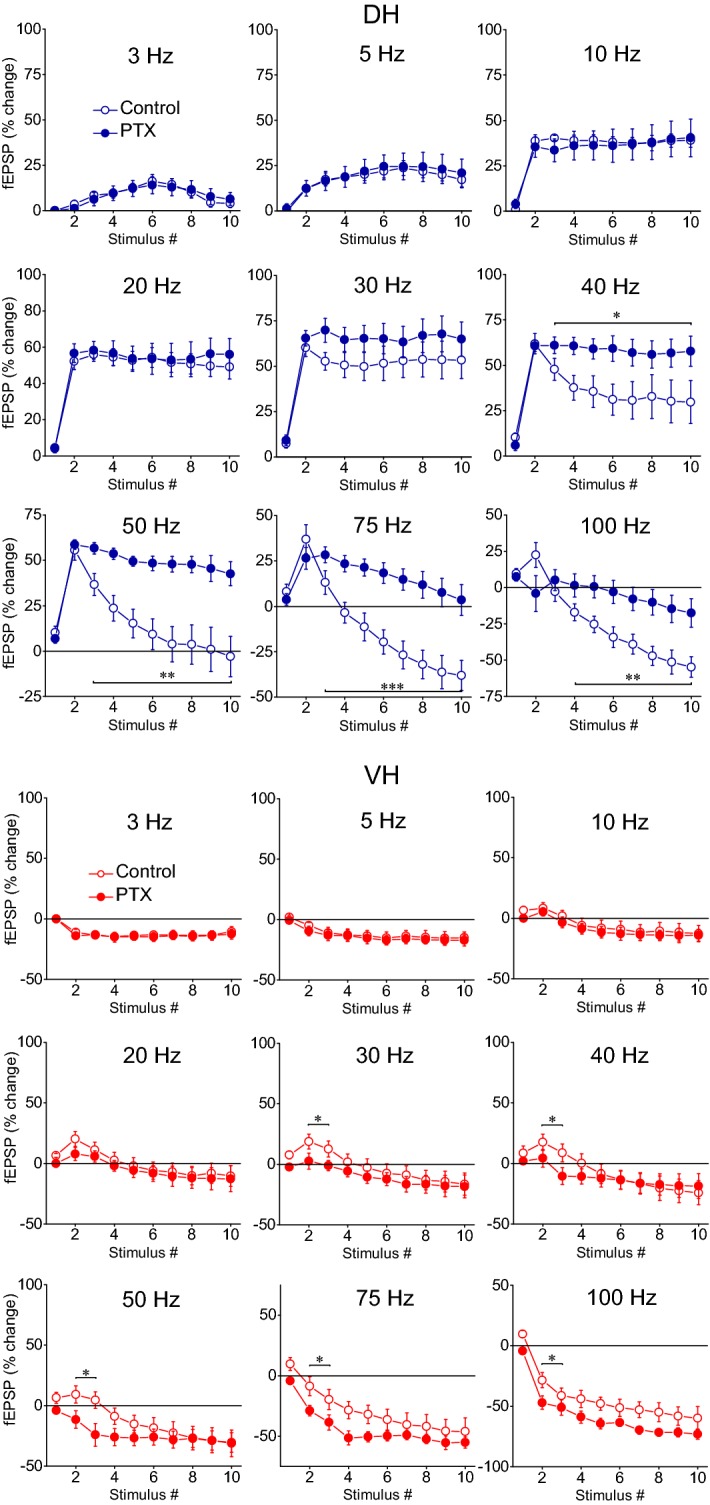
GABA_A_ receptor-mediated inhibition strongly modulates short-term synaptic plasticity in the DH but not in the VH. Short-term changes in fEPSP are shown for stimulation frequencies ranging from 3 to 100 Hz and they are plotted as a function of stimulus number. Stimulation current intensity was tuned to evoke suprathreshold fEPSP that produced a PS of 0.5–1 mV (see Fig. 8). Data were obtained from (slices/rats): 6/3 in DH and 6/5 in VH. Horizontal bars show the stimulation epoch in which significant drug effects were observed (paired t-test of individual responses along the train between experimental conditions). Asterisks denote the level of significance: *p < 0.05, **p < 0.01, ***p < 0.005

We performed a series of experiments using DH and VH slices obtained from eleven rats in which short-term plasticity was studied before and during perfusion of slices with the blocker of GABA_A_ receptor-associated channel picrotoxin (PTX). We used a small concentration of PTX (5 μM) in order to avoid the development of runaway excitation that could hamper the ability to measure PS responses, according to preliminary experiments we performed using higher drug concentrations. We found that reduction in synaptic inhibition induced significant frequency-dependent changes in short-term synaptic plasticity in the DH (n = 6/3) but produced very limited changes in the VH (n = 6/5) (Figs. 8, 9). Specifically, in the DH slices PTX consistently and significantly increased steady-state facilitation of fEPSP at 40–50 Hz (paired t-test of steady-state responses, *p *< 0.05 and *p *< 0.01 for 40 Hz and 50 Hz respectively) and eliminated steady-state depression of fEPSP at 75–100 Hz (paired t-test of steady-state responses, *p *< 0.01; additional statistical results are shown in Fig. 9). Therefore, the inhibition in the DH controls the short-term synaptic plasticity at ≥ 40 Hz and appears as a sufficient mechanism to switch between facilitation and depression of the CA3 input to CA1 circuit in this hippocampal segment. Strikingly, PTX did not cause any appreciable change in the steady-state synaptic response of the VH and fEPSP continued to be depressed at all stimulation frequencies in the disinhibited slices (paired t-test of steady-state responses, *p *> 0.05; Fig. 9). However, PTX significantly increased synaptic responses at the beginning of stimulation train at stimulation frequencies 30–100 Hz (paired t-test of 2nd and 3rd response in the train, *p *< 0.05). Thus, moderately suppressing GABA_A_ receptor-mediated inhibition in the VH transiently facilitates the synaptic response at the onset of stimulation but does not affect steady-state synaptic responses.

**Fig. 10:**
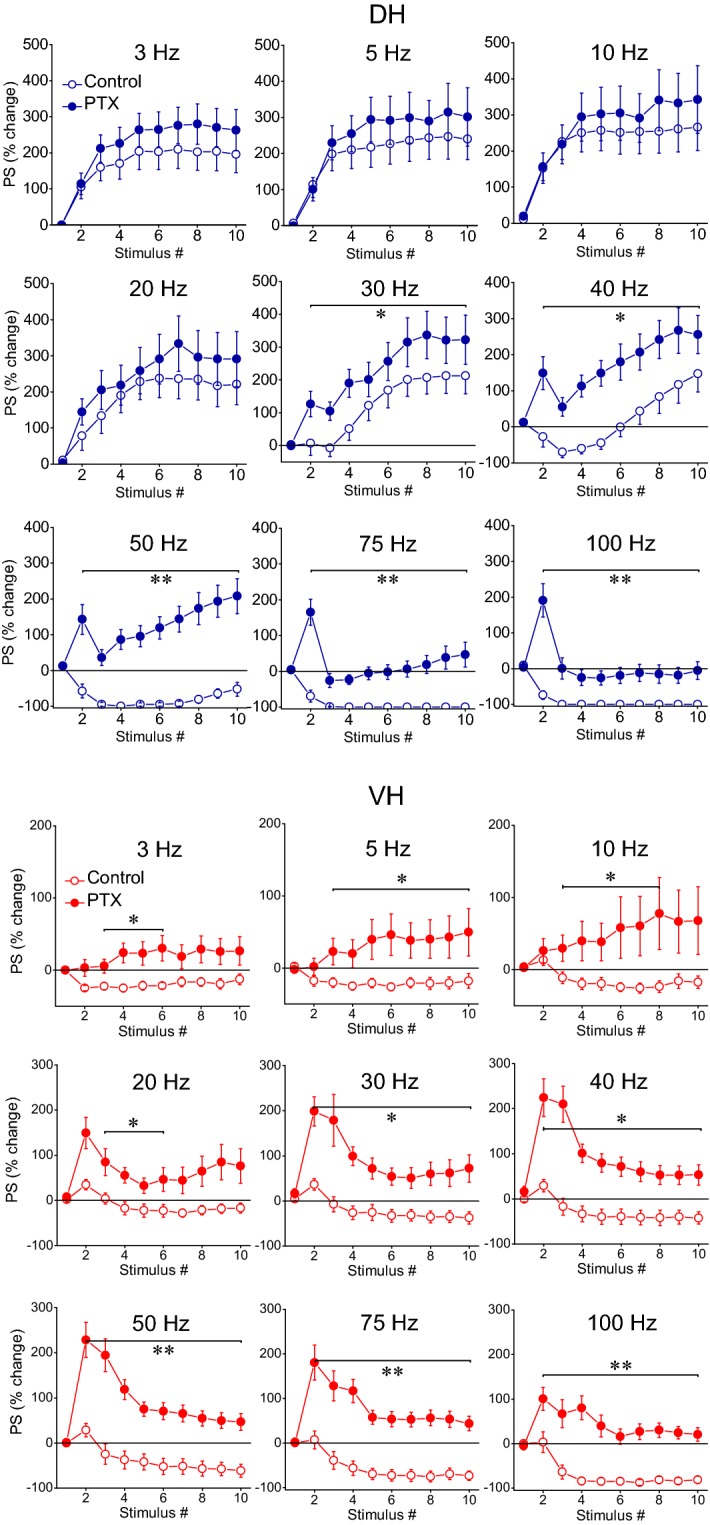
GABA_A_ receptor-mediated inhibition strongly modulates short-term dynamics of neuronal output in both hippocampal segments. Short-term changes in PS are shown for stimulation frequencies from 3 to 100 Hz and they are plotted as a function of stimulus number. Stimulation current intensity was tuned to evoke a PS of 0.5–1 mV. Data were obtained from (slices/rats): 16/8 in DH and 11/7 in VH. Horizontal bars show the stimulation epoch in which significant drug effects were observed (paired t-test of individual responses along the train between experimental conditions). Asterisks denote the level of significance: *p < 0.05, **p < 0.01

**Fig. 11:**
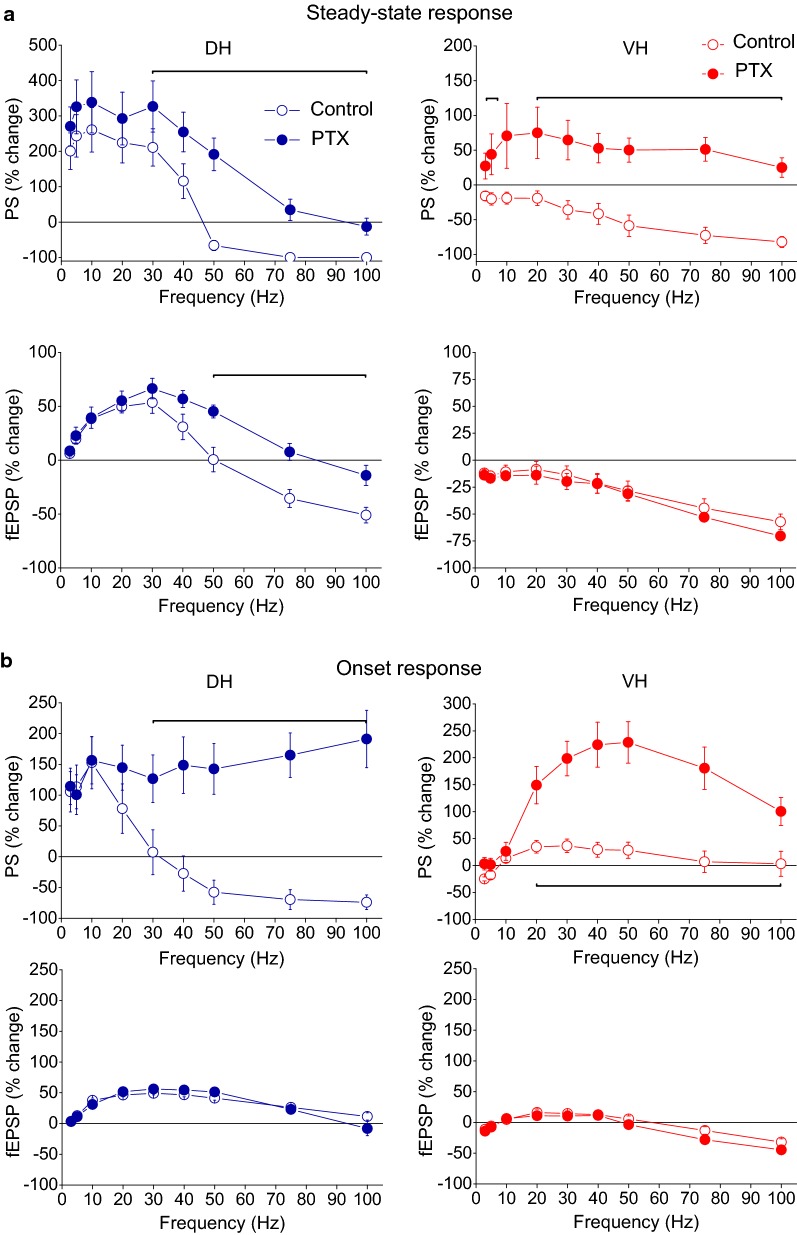
Steady-state (**a**) and onset responses (**b**) for synaptic input (fEPSP) and neuronal output (PS) in the DH and the VH under control conditions (open circles) and during blockade of GABA_A_ receptors by 5 μM picrotoxin (PTX, filled circles) plotted against stimulation frequency. Data were obtained with suprathreshold stimulation. Horizontal bars indicate the stimulation frequency range where statistically significant differences between control and drug condition were observed (paired t-test, the level of significance varies from *p *< 0.05 to *p *< 0.01)

Application of PTX produced robust effects on short-term dynamics of PS in both DH (n = 16/8) and VH slices (n = 11/7), and at a range of stimulation frequencies of 30–100 Hz in DH and 5–100 Hz in VH (Figs. 8, 10). Specifically, application of PTX in the DH slices significantly increased the steady-state facilitation (at 30–40 Hz) or eliminated the steady-state depression of PS (at 75–100 Hz) and transformed steady-state depression into strong steady-state facilitation at 50 Hz (paired t-test, *p *< 0.05 at 30–40 Hz, and *p *< 0.01 at 50–100 Hz; see also Fig. 10). In the VH, PTX transformed the steady-state depression of PS into steady-state facilitation across the range of stimulation frequencies from 5 to 75 Hz (but 10 Hz) and completely eliminated depression at 100 Hz (paired t-test, *p *< 0.05 for 5, 20, 30 and 40 Hz, and *p *< 0.01 for 50–100 Hz) (Fig. 11a); for comparison along the entire stimulation train (see Fig. 10). Markedly, stimulation of disinhibited slices transiently and strongly increased the amplitude of PS at the beginning of 30–100 Hz and 20–100 Hz stimulation in DH and VH respectively (i.e. onset response, Figs. 10, 11b; for particular statistical results (see Figs. 10, 11), indicating that a transient suppression of inhibition can permit the transmission of high frequency signals from the CA1 circuitry to its targets in a very reliable way. It is noted that the measurements under PTX were made after adjustment of the stimulation current so that the amplitude of PS was similar to that evoked under control conditions (0.8 ± 0.05 mV vs 0.78 ± 0.04 mV, n = 16 in DH and 0.85 ± 0.03 mV vs 0.84 ± 0.07 mV, n = 11 in VH), thus making the comparisons appropriate.

**Fig. 12:**
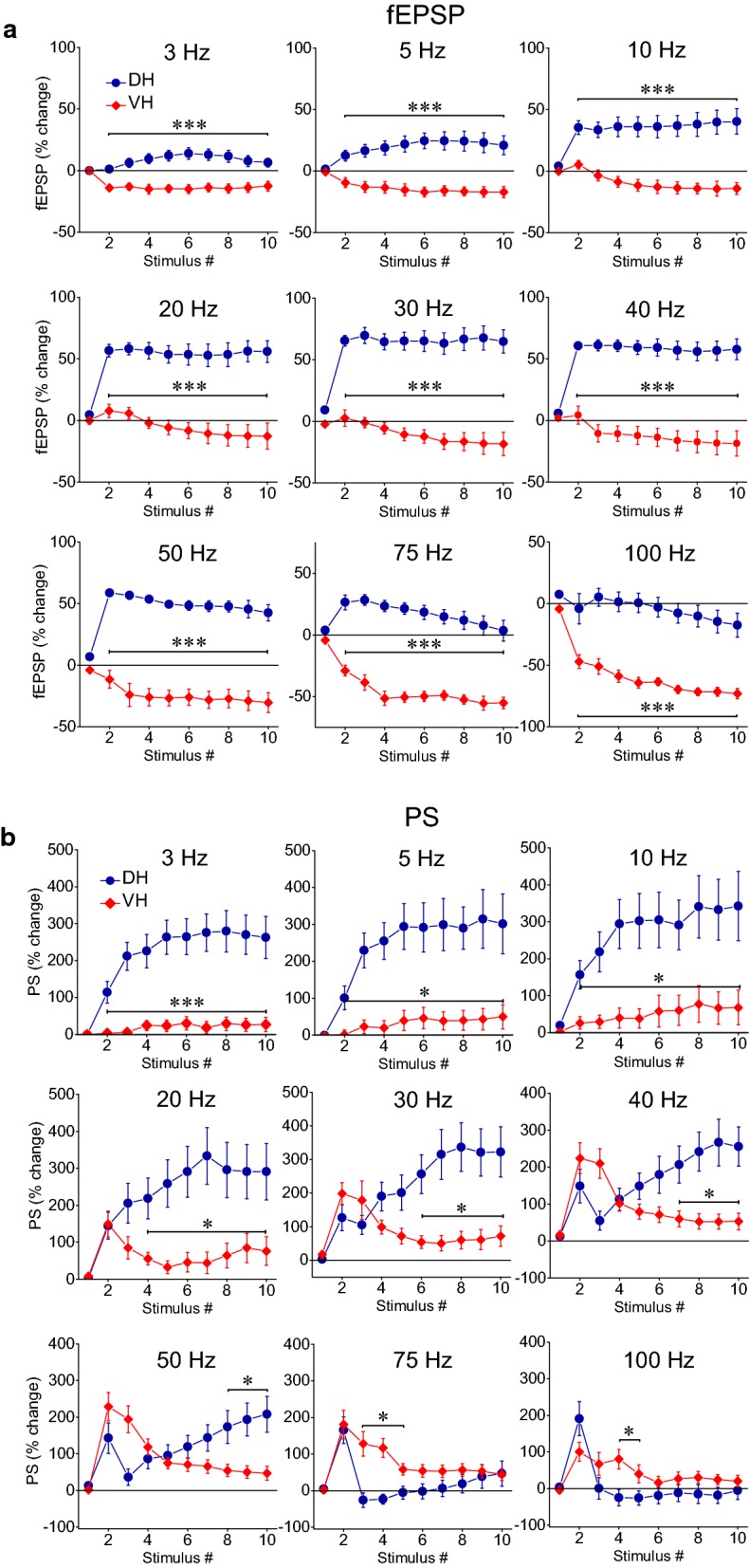
Short-term dynamics of fEPSP and PS induced by suprathreshold stimulation in disinhibited DH (circles) and VH slices (diamonds). Data presented in **a** are the same as the data presented in Fig. 10 (PTX), and data presented in **b** are the same as the data shown in Fig. 11 (PTX). In this figure however data from DH and VH are plotted on the same graphs for facilitating comparisons of disinhibited responses between the two hippocampal segments. Statistically significant differences of responses evoked along the entire stimulation train, between the two hippocampal segments are shown by horizontal bars (MANOVA along entire train of responses and independent t-test of individual responses along the train, *p < 0.05, ***p < 0.005). Note that partial disinhibition produced by 5 μM PTX widens the differences in short-term synaptic plasticity between DH and VH by strongly increasing facilitation or reducing depression of fEPSP in DH (compare data in **a** with data shown in Fig. 3b). Also, the higher steady-state depression of PS observed in DH than in VH under control conditions is reversed into increased steady-state facilitation in DH at the stimulation frequency of 50 Hz (compare data in **b** with data shown in Fig. 5**a**)

Comparisons of short-term dynamics of synaptic input (fEPSP) induced under PTX between DH and VH showed that disinhibition strongly widens the differences between the two hippocampal segments, compared with control conditions, at all stimulation frequencies (i.e. 3–100 Hz, compare Fig. 12a with Fig. 3b; details of statistical comparison are given in Fig. 12). Concerning neuronal output (PS), the most prominent effect of disinhibition is that the higher steady-state depression observed in DH vs VH under control conditions is reversed into higher steady-state facilitation of PS in DH vs VH at the stimulation frequency of 50 Hz (compare Fig. 12b with Fig. 5a; details of statistical comparison are given in Fig. 12). Individual data points of the drug effects on steady-state responses (fEPSP and PS) are shown in Additional file 2: Figure S2.

### μ-Opioid receptors modulate short-term dynamic in the DH but not the VH

The previous results indicated that short-term dynamics of neuronal output in CA1 are powerfully controlled by GABAergic inhibition in both DH and VH, albeit with different frequency characteristics in the two hippocampal segments. Excitation of CA1 principal neurons is controlled by several classes of GABAergic interneurons many of which belong to so-called basket cells that innervate the perisomatic region of pyramidal cells [88, 89]. In the hippocampus, the inhibition is powerfully modulated by μ-opioid receptors (μ-ORs) [90–92], which are selectively expressed by a subtype of basket cells, namely parvalbumin-expressing (PV) cells, and activation of μ-ORs inhibits the release of GABA from PV cells [93, 94]. Interestingly, the density of PV cells in the stratum pyramidale is higher in the DH than in the VH [95], however, μ-ORs in the CA1 stratum pyramidale are more abundant in the VH than in the DH [96–98], leading to an interesting question as to whether μ-ORs modulate short-term dynamics differently in the two hippocampal segments.

**Fig. 13:**
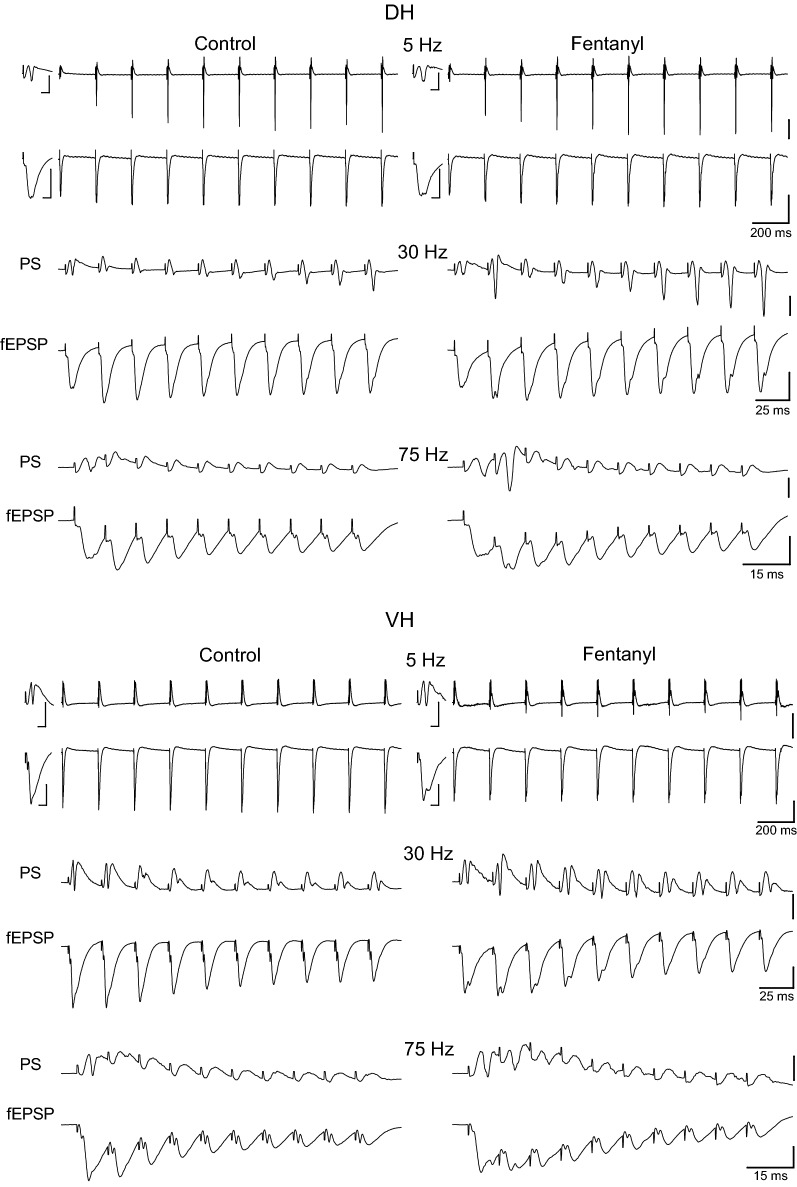
Examples of fEPSPs and PS evoked by three representative stimulation frequencies in a dorsal and a ventral hippocampal slice obtained before (Control) and during application of the agonist of μ-opioid receptors (μ-ORs) fentanyl (Fentanyl). Experiments were performed at stimulation current intensity that produced PS with amplitude of 0.5–1 mV. Single traces on the left of first PS and fEPSP traces (i.e. in the 5 Hz example) in each panel represent the first response of the 0.1 Hz train with which each frequency stimulation experimental procedure started; time calibration bars, 5 ms. Amplitude calibration bar for all records, 1 mV. Stimulation artifacts are truncated for clarity

**Fig. 14:**
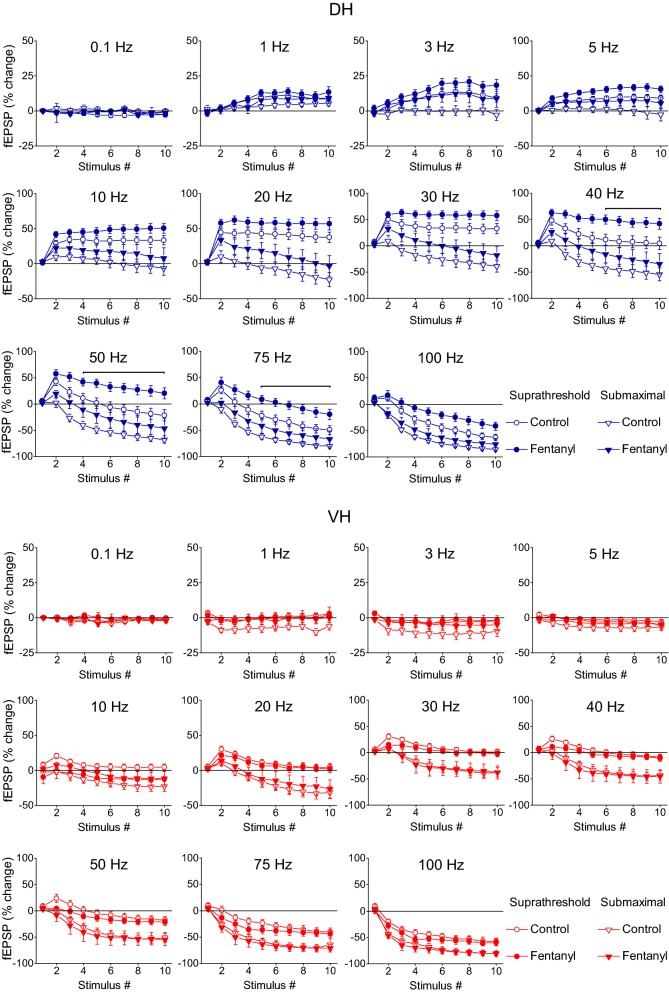
μ-ORs modulate short-term synaptic plasticity in the DH but not in the VH. Short-term changes in fEPSP induced by stimulation of varying frequency are plotted as a function of stimulus number. The effects of activation of μ-ORs by fentanyl were examined at two stimulation current intensities: suprathreshold (circles) and submaximal (triangles) that caused a PS with amplitude 0.1–0.5 mV and 75% of maximal value respectively. The bottom right diagrams in the two panels (upper, DH and lower, VH) illustrate the average values of fEPSP changes produced by the 8th–10th stimuli (steady state response) plotted as a function of stimulation frequency. Data shown in the two stimulation intensities (suprathreshold and submaximal) were obtained from (slices/rats): 9/8 and 7/6 in the DH and 7/6 and 8/7 in the VH. Statistically significant drug effects observed only on responses evoked by suprathreshold stimulation are shown by horizontal bars (paired t-test of individual responses along the train, *p *< 0.05)

**Fig. 15:**
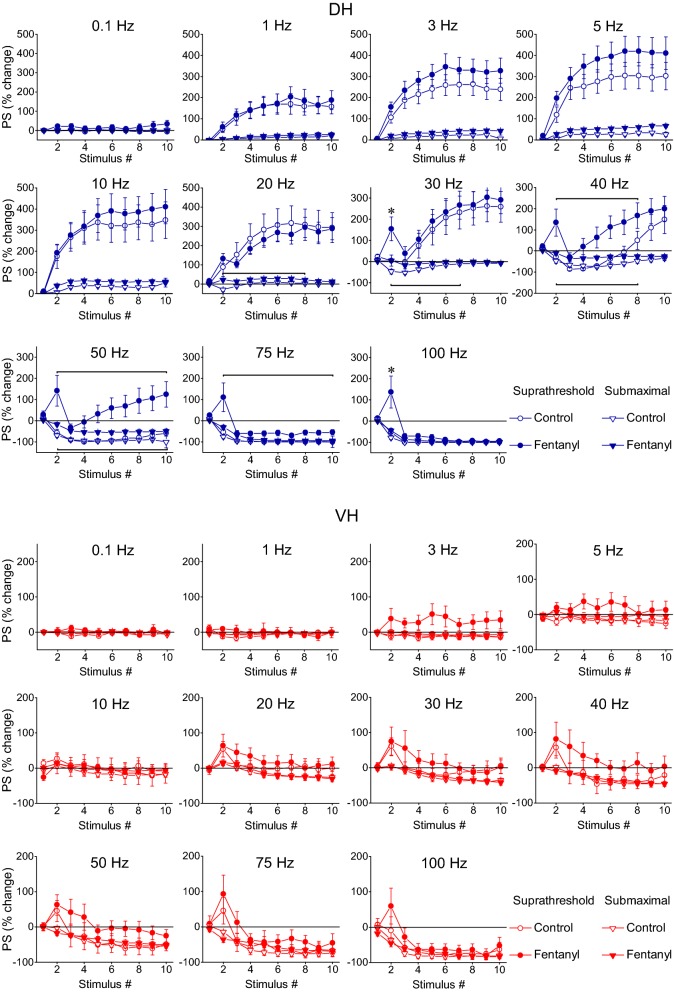
μ-ORs modulate short-term dynamics of neuronal output in DH but not in VH. Short-term changes in PS induced by frequency stimulation were obtained under control conditions (open symbols) and during application of fentanyl (filled circles). Two stimulation current intensities were used: suprathreshold (circles) and submaximal (triangles) that caused a PS with amplitude 0.1–0.5 mV and 75% of maximal value respectively. Percent changes are plotted as a function of stimulus number. The bottom right diagrams in the two panels (DH and VH) illustrate the average values of fEPSP changes produced by the 8th–10th stimuli (steady state response) plotted as a function of stimulation frequency. Data shown in the two stimulation intensities (suprathreshold and submaximal) were obtained from (slices/rats): 13/10 and 11/8 in the DH and 7/6 and 8/7 in the VH. Statistically significant drug effects observed on responses evoked by suprathreshold stimulation are denoted by horizontal bars or an asterisk (paired t-test of individual responses along the train, *p *< 0.05). Bars above and below the data curves denote significant drug effects on responses induced by suprathreshold and submaximal stimulation respectively

**Fig. 16:**
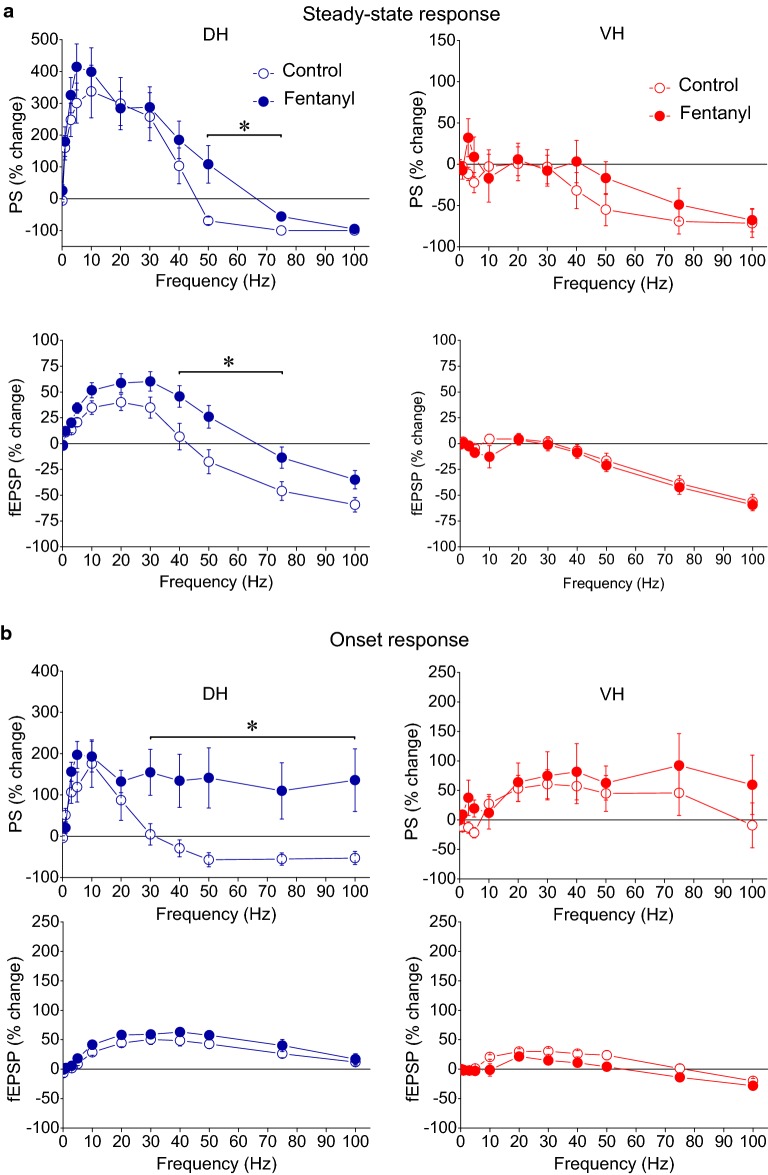
Steady-state (**a**) and onset responses (**b**) for synaptic input (fEPSP) and neuronal output (PS) in DH and VH under control conditions (open circles) and during activation of μ-Ors by fentanyl (filled circles) plotted against stimulation frequency. Data shown were obtained with suprathreshold stimulation. Horizontal bars above curves indicate the frequency range were statistically significant differences were observed between control and drug condition (paired t-test at *p *< 0.05). Note that symbols in **a**, for VH–fEPSP, showing results in the two conditions are overlapped between each other

We proceeded to investigate this question examining the effects of the specific agonist of μ-ORs fentanyl (10 μM) on DH and VH slices obtained from thirteen rats using suprathreshold and submaximal stimulation intensities. Examples of recordings are shown in Fig. 13. We find that fentanyl significantly influenced short-term dynamics of synaptic input and neuronal output in the DH; strikingly, however, activation of μ-ORs did not affect the dynamics of either fEPSP or PS in the VH (Figs. 14, 15, 16). Specifically, in the DH fentanyl significantly changed short-term dynamics of fEPSP (n = 9/8) and PS (n = 13/10) evoked by suprathreshold stimulation and at stimulation frequencies from 40 to 75 Hz as shown in Figs. 14, 15 (see figures and their legends for more statistics). Most characteristically, fentanyl reversed steady-state depression of both fEPSP and PS into steady-state facilitation at 50 Hz (paired t-test of average 8th–10th responses between conditions, *p *< 0.05) (Fig. 16a). With submaximal stimulation, fentanyl did not consistently influence fEPSP in DH (n = 7/6), thought it affected short-term dynamics of PS (n = 11/8) at stimulation frequencies 20-50 Hz (for more details see Fig. 15). Interestingly, the onset PS responses in the DH evoked with suprathreshold stimulation were consistently and significantly affected by fentanyl at stimulation frequencies of 30–100 Hz (paired t-test, *p *< 0.05) (Fig. 16b). Thus, fentanyl produced a very transient and robust enhancement of PS at the beginning of pulse sequence. Fentanyl did not affect onset synaptic responses (fEPSP) in the DH at any stimulation intensities. Remarkably, fentanyl did not significantly affect the short-term dynamics of either fEPSP or PS in the VH with either suprathreshold or submaximal stimulation (n = 7/6 and n = 8/7 respectively) (paired t-test of onset and steady-state responses, *p *> 0.05; Figs. 13, 14, 15, 16). Individual data points of the drug effects on steady-state responses (fEPSP and PS) are shown in Additional file 3: Figure S3.

## Discussion

These experiments show that DH presents an increased range of short-term dynamics both in synaptic input (fEPSP) and neuronal output (PS), ranging from strong response facilitation to its complete depression. Instead, the input and output responses in the VH are mostly depressed, and the VH network only very transiently allows a moderate output facilitation at the onset of stimulation train. Importantly, inhibition plays a crucial role in modulating short-term synaptic plasticity in the DH but not the VH, and strongly controls neuronal output gain in both hippocampal segments. Furthermore, μ-ORs significantly modulate short-term dynamics of both input and output only in the DH.

The large dorsoventral differences in short-term dynamics could be apparently attributed, to an appreciable extent, to the higher synaptic facilitation of the DH compared with the VH. It has been previously established that paired-pulse synaptic facilitation, a form of short-term plasticity [82], greatly differs between the DH and the VH CA3-CA1 synapses [35, 38, 48, 50, 69, 76, 78, 99]. This difference has been tentatively attributed to constitutive properties of synapses which are perhaps mainly concerned with the probability of transmitter release, though this issue is not yet fully resolved [40]. For instance, a reduced constitutive transmitter release probability could lead to increased synaptic facilitation and conversely, synapses with increased intrinsic probability of transmitter release will mostly depress upon repetitive activation because of depletion of neurotransmitter [100]. Besides, other presynaptic and postsynaptic mechanisms may also significantly contribute to determining the specific scores of short-term synaptic plasticity in the DH and the VH. These mechanisms may include inactivation of presynaptic calcium channels or release sites and saturation or desensitization of postsynaptic receptors [82]. Furthermore, postsynaptic mechanisms of non-linear summation of synaptic potentials may be particularly relevant for explaining the dependence of short-term synaptic plasticity on the intensity of presynaptic activation, especially whenever a relatively large population of synapses is under consideration [79], as is the case in the present study. It is also reasonable to assume that mechanisms that contribute to short-term synaptic plasticity will significantly influence neuronal spiking activity, in that, increased synaptic facilitation may lead to an increased output gain and depressing synapses could be expected to result in depression rather than facilitation of neuronal excitation. Accordingly, the strong facilitation of PS in the DH could be due, to a certain extent, to the increased facilitation shown by the DH synapses. However, a partial, yet important diversification of the mechanisms controlling synaptic input and neuronal output is suggested by some present data obtained from both hippocampal segments. For instance, in the DH the frequency-dependent profile of output responses at the beginning of stimulation is remarkably different from that of synaptic responses. Also, high-frequency stimulation induces a higher depression in PS compared with fEPSP. Even more striking is the unique pattern of neuronal excitation induced by 30–40 Hz stimulation that contrasts the corresponding pattern of synaptic responses in the DH.

We find that GABA_A_ receptor-mediated inhibition profoundly modulates both input and output dynamics in the CA1 microcircuit with, however, marked differences between the DH and the VH. In the DH, inhibition powerfully controls short-term synaptic plasticity in a frequency-dependent manner, limiting steady-state facilitation and depression of synaptic responses at 30–50 Hz and higher stimulation frequencies respectively. Strikingly, partial reduction of inhibition by low concentration of PTX does not significantly affect the depression of synaptic responses in the VH, suggesting that other mechanisms, presumably concerning with intrinsic synaptic properties may play a major role in depressing synaptic responses in VH and emphatically diversifying the mechanisms of short-term dynamics between input and output. Despite the striking dorsoventral differences in the action of inhibition in synaptic plasticity, inhibition powerfully controls steady-state neuronal spiking activity in both DH and VH. Furthermore, inhibition profoundly controls neuronal spiking activity at the onset of 20–100 Hz stimulation in both hippocampal segments. Interestingly, the effect of PTX to strongly facilitate neuronal output at the onset of stimulation occurs in the absence of drug effects on fEPSP. These observations suggest that different types of inhibition modulate neuronal output at different stages of stimulation.

A major mechanism that controls neuronal excitation in the hippocampus is the GABA_A_ receptor-mediated inhibition exerted by a variety of inhibitory interneuron circuits that target to the somatic and perisomatic region of principal neurons [101]. These inhibitory interneurons are activated either by the same input that activates principal neurons (feed-forward inhibition) or by the principal neurons following their own activation (feed-back inhibition) [88]. A fundamental consequence of the functional diversification between different circuits of interneurons is the existence of alternative modes of time and frequency-dependent modulation of neuronal firing during repetitive presynaptic activation [85, 86]. Importantly, the different properties of distinct types of inhibitory interneurons include the properties of short-term plasticity of the excitatory synapses that activate interneurons [83]. Actually, the pattern of inhibitory actions found here is consistent with the previously demonstrated pattern of successive actions of distinct interneuron circuits during repetitive stimulation of Schaffer collaterals [83]. In this study it was shown that application of a short stimulation train of pulses to CA1 stratum radiatum first elicits a strong somatic inhibition and then inhibition shifts towards more apical dendritic regions, suggesting the distinction of two populations of interneurons with different short-term plasticity properties of their receiving excitatory synapses, called onset-transient and late-persistent interneurons respectively [83]. Thus, the depression of PS at the onset of stimulation seen in the present study may be attributed to the action of “onset-transient” interneurons, while the steady-state depression of PS produced at later stages of 50–100 Hz stimulation, which is accompanied by robust control of fEPSP, could be attributed to the action of “late-persistent” interneurons. Accordingly, in the DH, inhibition targeted primarily on the somatic region controls response at the beginning of repetitive presynaptic activation, while dendritic inhibition is important in modulating synaptic dynamics that, then influence output dynamics.

Neuromodulation is pivotal for controlling neural information processing [72, 102]. For instance, μ-OR plays an important role in integration of distinct afferent inputs to CA1 field [103] The present results show that μ-ORs significantly modulate short-term dynamics of both synaptic input and neuronal output in the DH. However, we found no role of μ-ORs in the VH. The μ-ORs strongly enhance neuronal excitability in the hippocampus by reducing the release of GABA from pyramidal cell-targeting PV interneurons [91, 93], which are more abundant in the DH than in the VH [95], therefore explaining the increased action of μ-ORs on short-term dynamics in the DH. However, in an attempt to reconcile the higher action of μ-ORs with their lower number in the DH compared with the VH [96–98], we hypothesize that either μ-ORs can be substantially expressed by multiple cell types in the VH or that the role of PV cells on short-term dynamics is reduced in the VH, perhaps because their axonal arborization is reduced in the VH. Alternatively, it may be needed an increased activation for the PV cells in order to effectively release GABA and their control on pyramidal cell activity in the VH to be observed. This activity level may not be achieved under the experimental conditions used in the present study and therefore a possible action of μ-ORs could not be detected, in the hippocampus. Overall, μ-ORs play a significant role in distinguishing information processing between the DH and the VH.

### Implications for DH and VH functioning

The increased short-term dynamic range that the local CA1 circuit of the DH presents is expressed by its ability to integrate synaptic inputs and facilitate the output of principal neurons across a wide range of frequencies of presynaptic activity while it also can depress the output at relatively high frequencies. In other words, DH displays increased sensitivity to the frequency of presynaptic activity and can amplify or suspend the local circuitry output depending on the input frequency. Instead, the local network of VH presents a pattern of responses that consists of a stereotypical depression of output at a wide range of afferent input frequencies. Nevertheless, CA1 neurons in the VH transiently increase their activation at the onset of a sequence of afferent inputs of preferably moderate intensity. Virtually, this kind of response represents an ability of the local network to reliably detect and signal incoming patterns of activity, without being engaged in transmitting activity in a sustainable way. Such an ability of the VH may be particularly associated with the mechanisms underlying the specific functions of the VH. For instance, recent data has shown that VH can very reliably detect the coincidence of short bursts of presynaptic activity with beta adrenoceptor activity, that signal behaviorally important events, thereby strengthening its synaptic connections [52]. In addition, the VH presents an increased ability to initiate a memory-related network activity (i.e. sharp waves-ripples) [104] that requires detection of small transient increases in synaptic activity and neuronal excitability such as those observed at the start of repetitive stimulation in the present study [105, 106]. Hence, it can be assumed that the circuitry of the ventral segment of the hippocampus is responsible mainly for the signaling of arrival of afferent activity patterns at CA1 whereas the dorsal counterpart takes on the labor to steady transmit amplified information to other brain regions. A particularly representative example of these distinct putative roles of the two hippocampal segments is expressed by their conspicuously different patterns of output responses during 30–40 Hz afferent stimulation. Thus, the CA1 firing activity augments in the VH and decreases in the DH at the onset of stimulation train while at immediately later stages the neuronal activity is decreased in the VH and intensively enhanced in the DH. It is noted that activity propagation from the hippocampus to extra hippocampal targets occurs following activation of mainly DH CA3 and CA1 neurons [70].

Furthermore, the finding that modulation of output gain differs from that of input in the DH, over time and frequency, but both parameters are similarly modulated in the VH (Figs. 2, 3, 4) may be suggestive of greater layer-specific information processing in the DH compared with the VH. Nevertheless, modulation of presumably feed-back inhibition allows for an independent control of neuronal output gain in the VH; instead, in the DH a rather concerted inhibitory control of input and output (likely ascribed to feed-forward and feed-back inhibitory circuits respectively) is revealed. Also, it is particularly interesting that comparable results in short-term synaptic plasticity have been recently found in the transverse (tangential) and radial axis of the DH CA1 field [107], suggesting that information processing is distributed across all axes of the hippocampal network.

In addition to process and forward neural information to other brain structures, the hippocampus can selectively retain information in its endogenous network by mechanisms of lasting synaptic strengthening [108]. Many patterns of neuronal activity required to induce long-term potentiation, at least experimentally, firstly cause short-term changes in synaptic transmission and postsynaptic activation. Usually, these short-term changes consist of an increased postsynaptic depolarization [68, 109]. Importantly, facilitated postsynaptic firing that may result in response to a presynaptic burst input, such as that induced by 1–40 Hz in the present study, can crucially assist in the induction of long-term potentiation [47, 69, 110, 111]. Accordingly, the increased short-term facilitation of neuronal firing seen in the DH, besides its role in information transmission, may represent a means by which neural information is favorably embodied in the neuronal circuitry of the DH by way of synaptic strengthening.

## Conclusions

This study demonstrates that the CA1 circuitry of the DH displays a higher dynamic range of short-term plasticity of synaptic input and neuronal output mainly in virtue of the high scores of signal facilitation. On the contrary, the mostly depressing VH network selectively permits an enhancement of neuronal output at the beginning of a sequence of presynaptic activations. Furthermore, GABA_A_ receptor-mediated inhibition plays a crucial role in suppressing synaptic input and neuronal spiking activity induced by high-frequency presynaptic activity in the DH, but controls neuronal excitation without affecting short-term plasticity of synaptic inputs in the VH, at least under the experimental conditions used in this study. Physiologically, a role in regulating GABAergic inhibition can be ascribed to μ-ORs in the DH but it should be attributed to other mechanisms in the VH. Thus, inhibitory mechanisms appear to provide higher network flexibility to DH compared with VH. Taken together, these findings suggest that the distinct properties of the opposite hippocampal segments regarding the dynamic ranges of short-term input/output plasticity and the frequency filtering characteristics may provide the VH with the assignment of detecting and transient signaling of the appearance of afferent activity patterns while the DH appears to subserve the sustained signaling and broadcasting of amplified neural information to extra hippocampal structures in a frequency-dependent manner.

## Additional files

**Additional file 1: Figure S1.** Scatter plots illustrating individual data points of steady-state responses (fEPSP, PS) obtained from DH and VH. Short-term changes in fEPSP were induced with subthreshold, suprathreshold and submaximal stimulation current intensity while changes in PS were induced with suprathreshold and submaximal stimulation intensity. Percent changes of fEPSP and PS are plotted against stimulation frequency.

**Additional file 2: Figure S2.** Scatter plots illustrating individual data points of steady-state responses (fEPSP, PS) obtained from DH and VH under control conditions and under perfusion of slices with 5 μM PTX. Responses were recorded following suprathreshold stimulation current intensity.

**Additional file 3: Figure S3.** Scatter plots illustrating individual data points of steady-state responses (fEPSP, PS) obtained from DH and VH under control conditions and under perfusion of slices with 10 μM fentanyl. Responses were evoked by suprathreshold and submaximal stimulation current intensity.

## Data Availability

All the datasets generated and analyzed during this study are kept in the Physiology lab, Dept of Medicine, University of Patras and they are available from the corresponding author on reasonable request.

## Abbreviations

ANOVA: one-way analysis of variance
DH: dorsal hippocampus
fEPSP: field excitatory postsynaptic potential
MANOVA: multivariate general linear model
μ-ORs: μ-opioid receptors
PS: population spike
PTX: picrotoxin
PV: parvalbumin
VH: ventral hippocampus
