# Supplementary figures and images for: Short-term dynamics of input and output of CA1 network greatly differ between the dorsal and ventral rat hippocampus
Source: BMC Neurosci. 2019 Jul 22;20:35. doi: 10.1186/s12868-019-0517-5 (PMC6647178; doi:10.1186/s12868-019-0517-5)

# DH

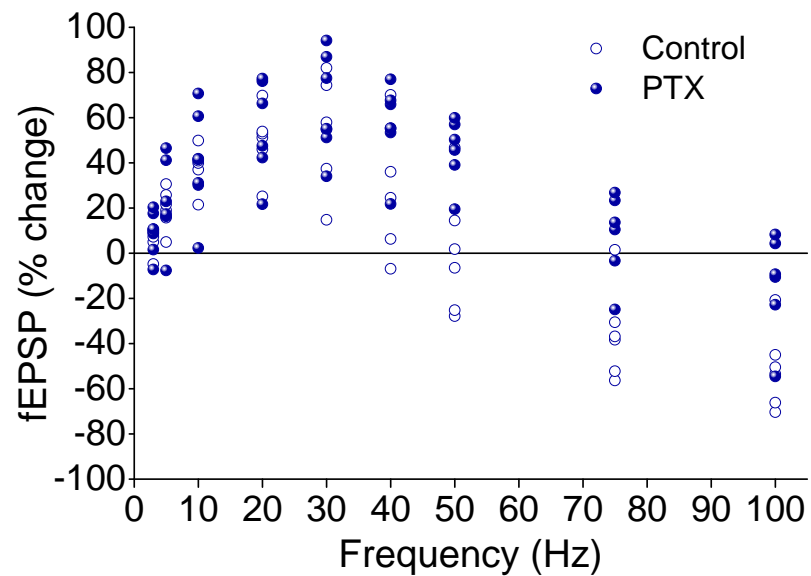

# VH

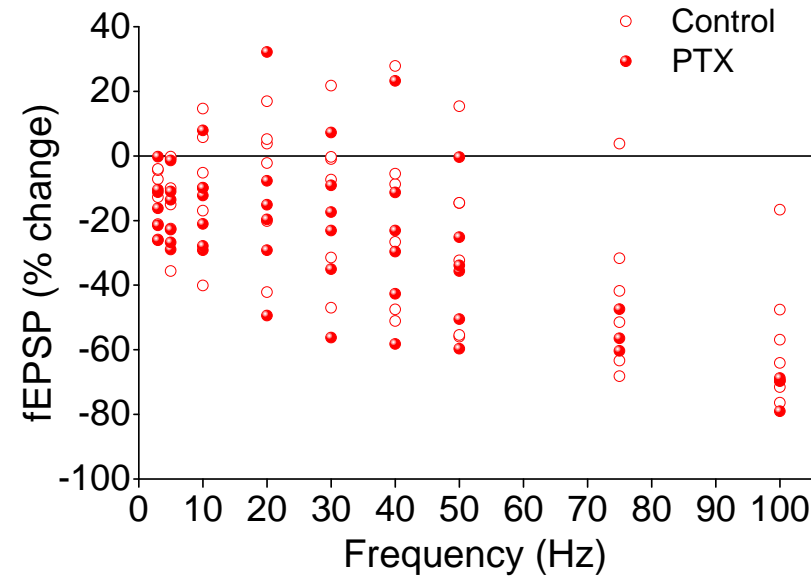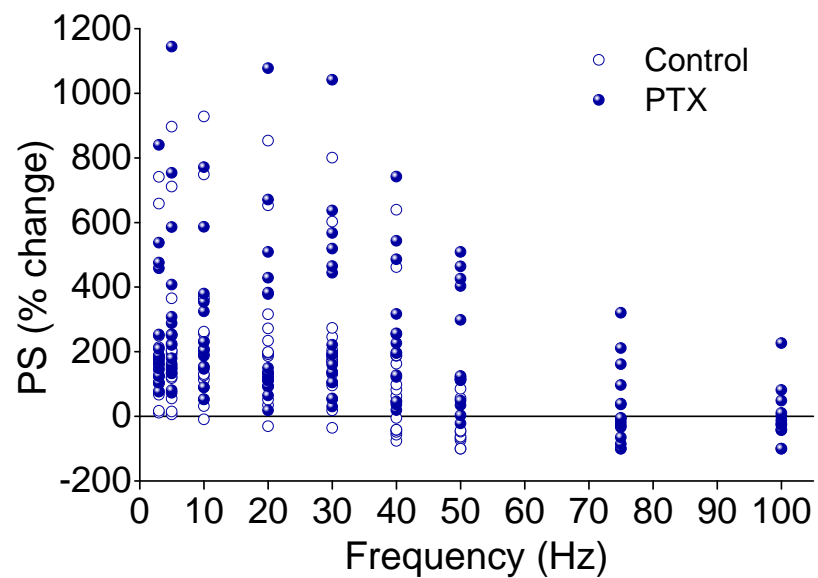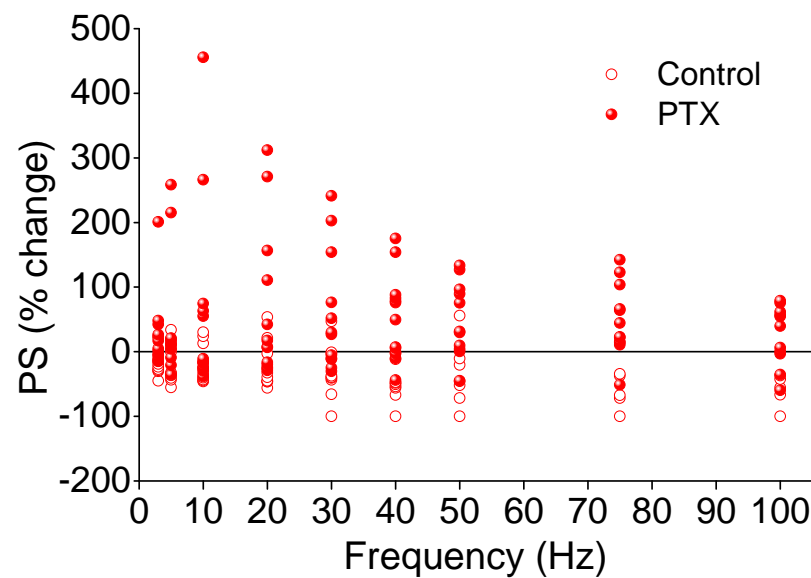

Supplement: Supplementary file 2 — Additional file 2: Figure S2. Scatter plots illustrating individual data points of steady-state responses (fEPSP, PS) obtained from DH and VH under control conditions and under perfusion of slices with 5 μM PTX. Responses were recorded following suprathreshold stimulation current intensity. [file 12868_2019_517_MOESM2_ESM.pdf]
